# Inflammasome activation under high cholesterol load triggers a protective microglial phenotype while promoting neuronal pyroptosis

**DOI:** 10.1186/s40035-023-00343-3

**Published:** 2023-03-09

**Authors:** Cristina de Dios, Xenia Abadin, Vicente Roca-Agujetas, Marina Jimenez-Martinez, Albert Morales, Ramon Trullas, Montserrat Mari, Anna Colell

**Affiliations:** 1 Department of Cell Death and Proliferation, Institut d’Investigacions Biomèdiques de Barcelona, Consejo Superior de Investigaciones Científicas (CSIC), Institut d’Investigacions Biomèdiques August Pi I Sunyer (IDIBAPS), Barcelona, Spain; 2 Centro de Investigación Biomédica en Red Sobre Enfermedades Neurodegenerativas (CIBERNED), Madrid, Spain; 3 Department of Biomedicine, Faculty of Medicine, Universitat de Barcelona, Barcelona, Spain; 4 Present Address: Department of Biochemistry and Molecular Biology, Faculty of Pharmacy, Universidad de Sevilla., Instituto de Biomedicina de Sevilla (IBiS)-Hospital Universitario Virgen del Rocío/CSIC, Seville, Spain; 5 Present Address: Department of Clinical Immunology and Rheumatology, Amsterdam UMC, Amsterdam, Netherlands

**Keywords:** Neuroinflammation, Mitochondrial oxidative stress, Phagocytosis, Alzheimer’s disease, DAM signature, NLRP3

## Abstract

**Background:**

Persistent inflammatory response in the brain can lead to tissue damage and neurodegeneration. In Alzheimer's disease (AD), there is an aberrant activation of inflammasomes, molecular platforms that drive inflammation through caspase-1-mediated proteolytic cleavage of proinflammatory cytokines and gasdermin D (GSDMD), the executor of pyroptosis. However, the mechanisms underlying the sustained activation of inflammasomes in AD are largely unknown. We have previously shown that high brain cholesterol levels promote amyloid-β (Aβ) accumulation and oxidative stress. Here, we investigate whether these cholesterol-mediated changes may regulate the inflammasome pathway.

**Methods:**

SIM-A9 microglia and SH-SY5Y neuroblastoma cells were cholesterol-enriched using a water-soluble cholesterol complex. After exposure to lipopolysaccharide (LPS) plus muramyl dipeptide or Aβ, activation of the inflammasome pathway was analyzed by immunofluorescence, ELISA and immunoblotting analysis. Fluorescently-labeled Aβ was employed to monitor changes in microglia phagocytosis. Conditioned medium was used to study how microglia-neuron interrelationship modulates the inflammasome-mediated response.

**Results:**

In activated microglia, cholesterol enrichment promoted the release of encapsulated IL-1β accompanied by a switch to a more neuroprotective phenotype, with increased phagocytic capacity and release of neurotrophic factors. In contrast, in SH-SY5Y cells, high cholesterol levels stimulated inflammasome assembly triggered by both bacterial toxins and Aβ peptides, resulting in GSDMD-mediated pyroptosis. Glutathione (GSH) ethyl ester treatment, which recovered the cholesterol-mediated depletion of mitochondrial GSH levels, significantly reduced the Aβ-induced oxidative stress in the neuronal cells, resulting in lower inflammasome activation and cell death. Furthermore, using conditioned media, we showed that neuronal pyroptosis affects the function of the cholesterol-enriched microglia, lowering its phagocytic activity and, therefore, the ability to degrade extracellular Aβ.

**Conclusions:**

Changes in intracellular cholesterol levels differentially regulate the inflammasome-mediated immune response in microglia and neuronal cells. Given the microglia-neuron cross-talk in the brain, cholesterol modulation should be considered a potential therapeutic target for AD treatment, which may help to block the aberrant and chronic inflammation observed during the disease progression.

**Supplementary Information:**

The online version contains supplementary material available at 10.1186/s40035-023-00343-3.

## Background

Increasing evidence indicates that chronic neuroinflammation is a critical driving force in the progression of Alzheimer’s disease (AD), more than a mere contributor to the exacerbation of tissue damage [1]. The induction of the immune response through astrocytes and microglial cells is a double-edged sword that helps to remove disease-specific pathological structures like amyloid beta (Aβ) deposits and dying cells but can also lead to worsening of the pathology when these cells are abnormally activated. It is well established that microglia, the principal immunocompetent mediators in the brain, can display multiple reactive phenotypes that may differ with the stage and severity of the neurodegenerative process [2, 3]. However, the specific factors that regulate this complexity and promote the gain of neurotoxic functions of microglia are largely unknown. In mouse models of AD, specific transcriptomic patterns, referred to as microglial neurodegenerative (MGnD) or disease-associated microglia (DAM) signatures [4, 5], have been identified in subpopulations of microglia exposed to neuronal or myelin debris and associated with Aβ plaques. These transcriptional changes are regulated by the triggering receptor expressed on myeloid cells 2 (*TREM2*) and apolipoprotein E (*APOE*), two main risk genes in human AD, and lead to decreased expression of homeostatic genes together with an upregulation of genes involved in lysosomal, phagocytosis, and lipid metabolism pathways [4, 5]. Nonetheless, although the molecular characterization suggests a protective role, whether DAM/MGnD signatures are beneficial or detrimental is still under debate [6].

Inflammasomes are key players in the innate immune response, and function as signaling platforms that can sense both pathogen-associated and damage-associated molecular patterns (PAMPs and DAMPs, respectively) released during tissue damage [7, 8]. Although the exact composition of these multi-molecule complexes depends on the activator, they classically comprise a NOD-like receptor (NLR) that acts as a sensor, such as NLRP3 and NLRP1, the adaptor protein apoptosis-associated speck-like protein containing a CARD (ASC, also referred to as PYCARD) and the catalytic protein caspase 1 (CASP1) [8]. In particular, the activation of the NLRP3 inflammasome has been reported to take place in two steps: first, the priming that stimulates the expression of inflammasome components and cytokine proforms, and then, the activator signal required to induce inflammasome assembly [8]. Once assembled, the functional complex activates CASP1, which regulates the maturation of the proinflammatory cytokines interleukin-1β (IL-1β) and interleukin-18 (IL-18) and, under some circumstances, can trigger gasdermin D (GSDMD)-mediated pyroptosis [9, 10]. In AD, activation of NLRP3 inflammasome has been shown to contribute to pathology progression by decreasing the phagocytic capacity of microglia [11, 12]. Accordingly, its inhibition primes microglia to a neuroprotective phenotype, resulting in decreased deposition of Aβ [11, 12]. Nonetheless, despite the link with the neurodegenerative process, the regulatory mechanisms underlying inflammasome activation in AD are not fully deciphered yet. Former studies by Zhou et al. reported that induction of the complex is supressed when expression of the mitochondrial carrier voltage-dependent anion channel 2 (VDAC2) is silenced or mitophagy is stimulated, indicating the involvement of mitochondrial reactive oxygen species (ROS) [13]. Aβ and monomeric/oligomeric tau can directly interact with NLRP3 [14, 15]; however, at least in retinal pigment epithelial cells, Aβ-mediated oxidative stress has also been linked to inflammasome assembly, with the participation of mitochondria [16].

Mitochondrial impairment is recognized as a common early event in AD that precedes amyloid plaque deposition and cognitive deficits [17]. Intracellular Aβ can alter mitochondria function and promote ROS generation [18, 19], which is further exacerbated by cholesterol-mediated depletion of mitochondrial glutathione (GSH) [20]. In the same direction, using transgenic mice that express the chimeric mouse/human amyloid precursor protein (Mo/HuAPP695swe) and mutant human presenilin 1 (PS1-dE9) together with the sterol regulatory element-binding transcription factor 2 (SREBF2), we have shown that brain cholesterol enrichment accelerates and worsens AD pathology including neuroinflammation, by enhancing the mitochondrial oxidative stress elicited by Aβ [21]. Abnormal brain cholesterol homeostasis has consistently been related to AD, with different experimental studies showing that high cholesterol promotes Aβ synthesis and deposition [22–24]. We have further demonstrated that intracellular cholesterol enrichment affects Aβ clearance by impairing autophagy [25]. More recently, we have found that cholesterol rises in mitochondria of hippocampal neurons in late-stage AD brains [26]. This abnormal cholesterol content inhibits mitophagy, resulting in an aberrant increase of mitochondrial content [26]. Based on these results, we hypothesized that high cholesterol may potentiate neuroinflammation by perpetuating a vicious cycle of increased Aβ levels and oxidative stress, thus favoring the induction of the NLRP3 inflammasome. It has been reported that oxysterols can regulate NLRP3 inflammasome, although with mixed outcomes [27–29]. Remarkably, at least in blood-borne macrophages, the ability of oxysterols to downregulate inflammasome activity, and thus IL-1β production, seems to rely on its inhibitory effect on cholesterol synthesis [27]. However, apart from these data, little is known about the relationship between deregulated brain levels of cholesterol and the induction of inflammatory signaling pathways in AD. Here, we set out to investigate whether an increase in the intracellular cholesterol content can modify the inflammasome-mediated response in microglia and neuronal cells.

## Materials and methods

### Cell culture and treatments

SH-SY5Y human neuroblastoma cell line (ECACC, 94030304) and the mouse spontaneously immortalized microglia-9 (SIM-A9) cell line (ATCC-CRL-3265) [30] were cultured in Gibco™ DMEM/ F-12 with GlutaMAX™ (ThermoFisher, Waltham, MA, 31331028), supplemented with 10% fetal bovine serum (ThermoFisher, Waltham, MA, 10100139) and 5 μg/ml plasmocin™ (InvivoGen, San Diego, CA, ant-mpt). The culture medium of SIM-A9 cells was supplemented with 5% horse serum (Sigma-Aldrich, Saint Louis, MO, H1270). Embryonic cortical-hippocampal neurons from wild-type (WT) and SREBF-2 mice (B6;SJL-Tg(rPEPCKSREBF2)788Reh/J, RRID:IMSR_JAX:003311) were isolated on day 16–17 of pregnancy by trypsin digestion following a standard protocol [31]. Dissociated cells were grown in Neurobasal™ medium (ThermoFisher, 21103–049) supplemented with 2.5% (*v*/*v*) B27 supplement (ThermoFisher, 17504–001), 0.5 mM *L*-glutamine (Sigma-Aldrich, G7513) and 5 μg/ml plasmocinTM (InvivoGen, ant-mpt), and plated onto poly-*D*-lysine (Sigma-Aldrich, P6407)- and laminin (Sigma-Aldrich, L2020)-coated plates at a density of 2 × 10^5^ cells/cm^2^. Half of the culture medium was changed every 3 or 4 days. Over 95% of neuronal purity was confirmed by immunochemistry using antibodies targeting neuronal and glial markers. Experiments were performed at 7 to 10 days in vitro (DIV). All procedures involving animals and their care were approved by the ethics committee of the University of Barcelona and were conducted in accordance with institutional guidelines in compliance with national and international laws and policies.

Cell cholesterol enrichment was achieved by incubation with a cholesterol:methyl-β-cyclodextrin complex (CHO:MCD; containing 50 μg/ml of cholesterol) (Sigma-Aldrich, C4951) for 1 h followed by 4-h recovery. To induce inflammasome activation, cells were treated with 10 μg/ml lipopolysaccharide (LPS) from *Escherichia coli* O111:B4 (Sigma-Aldrich, L4391), 10 μg/ml *N*-acetylmuramyl-*L*-alanyl-*D*-isoglutamine hydrate (also known as muramyl dipeptide, MDP; Sigma-Aldrich, A9519), 5 mM ATP (Sigma-Aldrich, A2383), 150 μg/ml monosodium urate crystals (MSU; Santa Cruz Biotech., sc-202711), and oligomeric Aβ at the indicated times. Preincubation with 4 mM glutathione ethyl ester (GSHee) or with the cell-permeable caspase 1 inhibitor I (10 μM; Bachem, 4095744) was performed 30 min before treatment when indicated.

### Preparation of Aβ peptides

Human Aβ (1–42) hydrochloride salt (Bachem, Bubendorf, Switzerland, H-6466) was dissolved to 1 mM in hexafluoroisopropanol (HFIP; Sigma-Aldrich, 10522–8), aliquoted and stored at − 20 °C after HFIP evaporation. For oligomeric assembly, peptides were resuspended to 5 mM in DMSO by sonication, then diluted to 100 μM in phenol red-free DMEM and incubated at 4 °C for 24 h [32]. Presence of soluble oligomeric forms of Aβ was confirmed by Western blot [20] and electron microscopy (See Additional file 1: Fig. S1).

### Western blotting

Cells were lysed in RIPA lysis buffer with 2 mM phenylmethylsulfonyl fluoride, 1 mM sodium orthovanadate and protease inhibitor cocktail (Santa Cruz Biotech, Dallas, TX, sc-24948) for 30 min at 4 °C and then centrifuged at 16,000* g* for 15 min. Samples (25–50 μg of protein/lane) were resolved by sodium dodecyl sulfate–polyacrylamide gel electrophoresis and transferred to nitrocellulose membranes (Bio-Rad, Hercules, CA, 1704271). Blots were probed with rabbit monoclonal anti-caspase 1 (1:1000; Abcam, Cambridge, UK, ab179515), rabbit polyclonal anti-IL-1beta/IL-1F2 (1:1000; Novus Biologicals, Abingdon, UK, NB600-633), rabbit polyclonal anti-NLRP1/NALP1 (1:1000; Cell Signaling, Danvers, MA, 4990S), rabbit polyclonal anti-NLRP3/NALP3 (1:2500; Novus Biologicals, NBP2-12446), and mouse monoclonal anti-ACTB/actin (1:30,000; Sigma-Aldrich, A3853). After overnight incubation at 4 °C, bound antibodies were visualized using horseradish peroxidase-coupled secondary antibodies and the Clarity™ Western ECL Substrate (Bio-Rad, 1705061) or Clarity™ Max ECL western blotting substrate (Bio-Rad, 1705062) for low protein concentrations.

### Selfie quantitative reverse transcription PCR (RT-qPCR)

Selfie RT-qPCR that measures the absolute number of RNA transcripts per gene was performed as described by Podlesniy and Trullas [33]. Briefly, cells were extracted and diluted in the 100ST buffer (DireCtQuant, Lleida, Spain, DCQ100ST) following the manufacturer’s instructions. Then, a pre-annealing step was performed per duplicate, by mixing 2 μl of the sample with the same volume of 2.5 μM of the corresponding reverse primer (*Trem2* 5′-ctcggagactctgacactgg-3′; *Clec7a* 5′-gcactgcagcaaccactact-3′) at 70 °C for 5 min. Next, the reaction mixture containing 1 mM dNTP mixture, 10 U of the RiboLock RNase Inhibitor (Thermo Fisher Sci., EO0381) and 200 U of Maxima H Minus Reverse Transcriptase (Thermo Fisher Sci., EP0751) or 50% glycerol was retro-transcribed for 30 min at 60 °C followed by 5 min at 85 °C. Finally, 2.5 μM of the corresponding forward primer (*Trem2* 5′-tggaaccgtcaccatcactc-3′; *Clec7a* 5′-cttcaccttggaggcccatt-3′) was added and amplified by conventional RT-qPCR (5 min at 95 °C, followed by 49 cycles of 15 s at 95 °C, 25 s at 60 °C and 25 s at 72 °C), using SsoAdvanced Universal SYBR Green Supermix (Bio-Rad, 1725271). The number of transcripts per encoding gene was calculated as the fold change after subtracting the Ct values obtained from the sample containing glycerol.

### Gene array

A predesigned 384-well mouse innate and adaptive immune response genes (SAB Target List) panel (Bio-Rad, 10034519) was used following the manufacturer's instructions. Briefly, after isolating RNA with the TRIzol reagent (Thermo Fisher, 15596026), the corresponding cDNA was synthesized using the iScript advanced cDNA synthesis kit (Bio-Rad, 1708891). Once cDNA was obtained, the PCR reaction mix was prepared (SsoAdvanced universal SYBR Green supermix, Bio-Rad, 1725271) and added to the 384-well plate where all the primers were lyophilized.

### Quantification of CLEC7A by flow cytometry

Cells were adjusted to a concentration of 5 × 10^5^ cells/ml in PBS containing 0.5% BSA and incubated with the PE-conjugated CD369 (Clec7a, Dectin-1) monoclonal antibody (Thermo Fisher Scientific, 12–9856-42, RRID: AB_2572749) and the corresponding IgG2a-PE isotype control (Thermo Fisher Scientific, 12-4724-82, RRID: AB_470064) (dilution 1:20) for 20 min at 4 °C. Then, cells were washed with 3 ml PBS and finally suspended in 500 μl PBS. Fluorescence intensity was measured using a Beckman Coulter Cell Lab Quanta SC Flow. An isotype control IgG staining for each sample was performed in order to subtract background.

### Cell viability

Cell viability was assessed by quantifying lactate dehydrogenase (LDH) released into the culture media upon plasma membrane disruption. The assay was performed using the CyQUANT LDH Cytotoxicity Assay Kit (ThermoFisher, C20301) following the manufacturer’s instructions. In some cases, cell death was also evaluated by trypan blue exclusion staining.

### ROS determination

Oxidative stress was assessed by using the cell-permeant fluorogenic dye 2′,7′-dichlorofluorescin diacetate (DCFH-DA; Sigma-Aldrich, D6883). First, cells were incubated with 20 μM DCFH-DA for 30 min at 37 °C and after rinsing with Live Cell Imaging Solution (ThermoFisher, A14291DJ), the oxidized product 2′-7′dichlorofluorescein (DCF) was detected by fluorimetry (Ex 495 nm, Em 529 nm). Then, cells were stained with 0.5% crystal violet for 15 min and after washing 3 times with H_2_O, the dye was solubilized by 0.1% sodium dodecyl sulfate and measured by absorbance at 550 nm. DCF intensity of each sample was normalized to the corresponding absorbance values of the crystal violet.

### Cholesterol and GSH measurements

Cholesterol levels were determined fluorometrically using the Amplex Red Cholesterol Assay kit (Thermo Fisher Sci.; A12216). Samples (0.5 × 10^5^ cells) were extracted with chloroform:isopropanol:IGEPAL CA-630 (7:11:0.1) and centrifuged at 13,000 g for 10 min to remove insoluble material. The organic phase was dried under vacuum, dissolved in 1 × cholesterol reaction buffer, and analyzed following the guidelines provided by the supplier. To evaluate the intracellular distribution of the cholesterol load, cells were fixed in 4% paraformaldehyde (PFA) for 20 min at room temperature, and after washing three times, were incubated with the naturally fluorescent polyene antibiotic filipin III (0.25 mg/ml; Sigma-Aldrich, F4767) for 30 min. Images were acquired with a Zeiss Axiophot fluorescence microscope using a 40 × /1.3 N.A. objective. The ImageJ software [34] was used to calculate the corrected total cell fluorescence (CTCF) by applying the following formula: CTCF = Integrated Density − (Area of selected cell × Mean fluorescence of background readings). Mitochondria from SH-SY5Y cells were isolated by digitonin fractionation as described previously [35] and the GSH content was analyzed using the Glutathione Assay Kit (Sigma-Aldrich, CS0260-1 KT) following the manufacturer's instructions.

### CASP1 activation

The activation of the enzyme was analyzed in living cells using the FAM-FLICA® Caspase 1 Assay kit (ImmunoChemistry Tech., Davis, CA) following the manufacturer’s instructions. Cells were labeled with the fluorescent inhibitor probe FAM-YVAD-FMK at the indicated times. After subsequent washes (to allow any unbound FAM-FLICA to diffuse out of cells), nuclei were stained with Hoechst 33,342 (1 μg/ml) for 10 min at 37 °C and cells were examined under a Zeiss Axiophot fluorescence microscope using a 40 × /1.3 N.A. objective. From each condition, 4 non-overlapping images from the top, middle and bottom areas were randomly taken. The number of green-positive and Hoechst-positive cells was determined by using the automated particle counting tool of ImageJ [34].

### Enzyme-linked immunosorbent assay (ELISA)

Cell supernatants were centrifuged at 3000*g* for 10 min and concentrated using Corning® Spin-X® UF centrifugal concentrators (Corning Life Sciences, Tewksbury, MA, 431,482). The levels of IL-1β in the culture media of SIM-A9 and SH-SY5Y cells were measured using mouse and human IL-1β ELISA kits, respectively (Abcam, ab229440 and ab100562), according to the manufacturer’s instructions. In some cases, to evaluate the IL-1β encapsulated in extracellular vesicles (EVs), Triton X-100 was added to the supernatants at a final concentration of 1% and the mixture was allowed to stand for 10 min at room temperature prior to assay of secreted cytokines. Quantification of neurotrophic factors was assessed in cell culture supernatants using the Multi-Neurotrophin Rapid™ Screening ELISA Kit (Biosensis, Thebarton, SA, Australia, BEK-2231).

### Immunofluorescence

Cells were seeded into 8-well LabTek removable chamber slides (Thermo Scientific, 177402). After treatment, cells were fixed with 4% paraformaldehyde (PFA) for 15 min before permeabilization with 0.1% saponin in blocking buffer (1% fatty acid free BSA, 20 mM glycine in PBS) for 15 min at room temperature. For non-permeabilized TREM2 staining, saponin was omitted from all blocking and washing steps. Then, samples were incubated with primary antibodies (rabbit polyclonal anti-TREM2, Proteintech, 13483–1-AP, 1:100, overnight at 4 °C; rabbit polyclonal anti-GSDMD, Abbexa, abx340202, 1:250, 3 h at 37 °C), followed by 1-h incubation at room temperature with the secondary antibody Alexa Fluor 488-conjugated goat anti-rabbit IgG (Thermo Fisher Sci., A-11008, 1:400). From each well, 3 non-overlapping images from the top, middle and bottom areas were randomly taken using a Leica TCS SPE confocal laser scanning microscope (Leica Microsystems) with a 63 × /1.32–0.60 oil PH3 CS objective and a confocal pinhole set at 1 Airy unit.

### Pyroptosis assessment

To assess GSDMD-driven pyroptotic cell death, cells were lipotransfected with a plasmid expressing mNeoGreen-GSDMD, a gift from Dr. Derek W. Abbott (Case Western Reserve University School of Medicine, Cleveland, OH). Cells were seeded in m-Slide 8-well chambered coverslips treated with ibiTreat (Ibidi, 80826) to 60%–70% of confluence. Transfections were conducted using Lipofectamine 3000 (Thermo Fisher Sci., L3000) in opti-MEM medium. Forty hours after transfection, cells were exposed to the corresponding treatment and imaged. In each experiment, 2–3 fields/condition were selected for time-lapse imaging using an Andor Dragonfly spinning disk confocal microscope (Andor Technology, Oxford Instruments) equipped with a Zyla 4.2 PLUS sCMOS camera. Cells were incubated in a chamber with a 5% CO_2_ atmosphere at 37 °C throughout the experiment. Fluorescence images were acquired at regular intervals of 20 min, with the use of a 60 × /0.17 MI-oil plan fluor objective. Image acquisition started at the moment of stimulation. Cells were imaged for 8 h. Mock-treated cells were followed in parallel to ensure that imaging and staining procedures were not cytotoxic. Representative images and movies were extracted and edited using ImageJ software [34].

### Visualization of ASC oligomerization

To evaluate the presence of ASC oligomers, cells were transfected with the pLEX-MSC-ASC-GFP plasmid, a gift from Christian Stehlik (Addgene plasmid #73957; http://n2t.net/addgene:73957; RRID:Addgene_73957) [36]. Cells were seeded in 8-well LabTek removable chamber slides (Thermo Scientific, 177402) to 60%–70% of confluence. Transfections were conducted using Lipofectamine P300 (Invitrogen) in opti-MEM medium. Forty-eight hours after transfection, cells were exposed to the corresponding treatment. After treatment, cells were incubated with the CellMask™ Orange plasma membrane stain (1:10,000; Thermo Fisher Sci., C10045) and fixed with 4% PFA. The formation of ASC specks was assessed as the formation of reticulated structures, visualized with a Leica TCS SPE confocal laser scanning microscope (Leica Microsystems, Spain) using a 63 × /1.32–0.60 oil PH3 CS objective and a confocal pinhole set at 1 Airy unit. From each well, three non-overlapping images from the top, middle and bottom areas were randomly taken. The number of speck-positive cells was determined by manual counting using ImageJ [34].

### Phagocytosis assay

Microglial phagocytosis was evaluated by quantifying the degree of internalization of the fluorescent HiLyte Fluor 488-labeled Aβ1-42 peptide (AnaSpec, AS-60479-01). Cells were seeded in 8-well LabTek removable chamber slides. After treatment, 1 μM HiLyte Fluor 488-labeled Aβ peptides was added and incubated for 4 h at 37 °C in a 5% CO_2_ atmosphere. Then, cells were washed three times with PBS to arrest uptake, and plasma membrane/cytosol and nuclei were labeled with CellMask™ Orange stain (1:20,000; Thermo Fisher Sci., C10045) and DRAQ5™ (1:500; Thermo Fisher Sci., 62251), respectively, for 5 min. Finally, cells were fixed with 4% PFA and observed under a Leica TCS SPE confocal laser scanning microscope (Leica Microsystems) using a 63x/1.32–0.60 oil PH3 CS objective and a confocal pinhole set at 1 Airy unit. From each well, three non-overlapping images from the top, middle and bottom areas were randomly taken. The ImageJ software [34] was used to calculate the CTCF by applying the following formula: CTCF = Integrated Density − (Area of selected cell × Mean fluorescence of background readings).

### Statistical analysis

All results are expressed as mean ± SD. Statistical significance was examined using the unpaired, two-tailed Student’s *t*-test or one-way analysis of variance (ANOVA) followed by the Tukey–Kramer test for multiple comparison, performed using the IBM SPSS Statistics software (ver. 28.0, IBM Corp.). A value of *P* < 0.05 was considered statistically significant.

## Results

### Cholesterol enrichment stimulates the release of encapsulated  IL-1β after PAMP-mediated activation of the NLRP3 inflammasome in SIM-A9 microglia

**Fig. 1:**
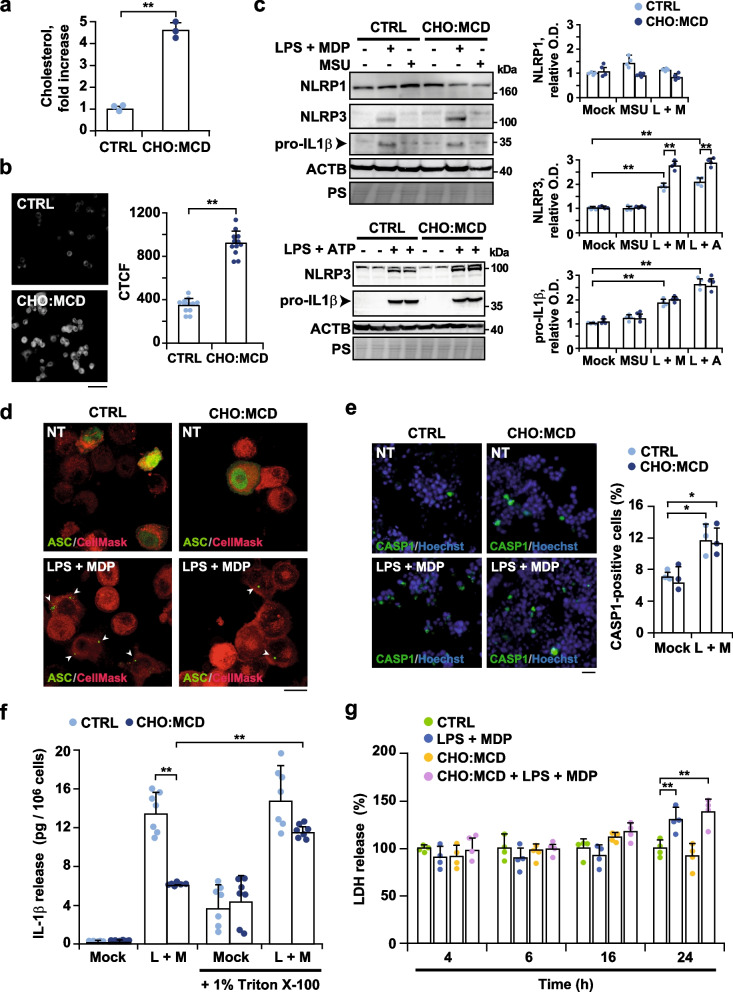
Activation of the NLRP3 inflammasome by PAMPs in SIM-A9 microglial cells after cholesterol enrichment. Cells were treated with the CHO:MCD complex for 1 h. Inflammasome was induced by co-incubation with LPS and MDP (L + M, 10 μg/ml each for 16 h), LPS (10 μg/ml for 16 h) and ATP (5 mM for 1 h) (L + A), or MSU (150 μg/ml for 4 h). **a** Total cholesterol levels of cellular extracts. (*n* = 3 independent experiments).** b** Filipin staining. Representative images from 3 independent experiments. Scale bar: 100 μm. Filipin staining was quantified as the CTCF of the green channel (*n* = 12). **c** Western blot analysis of NLRP1, NLRP3 and IL-1β in cellular extracts. (pro-IL-1β: IL-1β pro-form). ACTB/actin β and ponceau S (PS) staining were used as loading controls. Optical density (O.D.) values of the bands representing the specific protein immunoreactivity were normalized to PS staining (*n* = 4). **d** Representative confocal images of oligomeric ASC from 3 independent experiments. Cells were transfected with a plasmid encoding an ASC:GFP fusion protein and treated as indicated. Cells were counterstained with CellMask (cytosol/plasma membrane, red). Scale bar: 25 μm. **e** Representative fluorescence micrographs of CASP1-positive cells (green). The fluorescent CASP1 inhibitor was added during the inflammasome induction period (16 h). Nuclei were stained with 1 μg/ml Hoechst 33342. Scale bar: 100 μm. Data in the graph are expressed as % of CASP1-positive cells (green) over total Hoechst-stained cells (blue). (*n* = 3 independent experiments). **f** Levels of IL-1β in the cell culture supernatants. Values were normalized to the total protein content of the corresponding cellular extracts. In some cases, to assess the presence of IL-1β encapsulated in EV, supernatants were incubated with 1% Triton X-100 (*n* = 6–7 independent experiments). **g** Cell death by the LDH assay. Results are expressed as % to the untreated control values (*n* = 6 independent experiments). Two-tailed Student's *t*-test (**a** and **b**) and one-way ANOVA followed by the Tukey–Kramer test (**c**-**g**) were applied to calculate statistical significance (*P ≤ 0.05, **P ≤ 0.01). See Additional file 1: Fig. S5 for uncropped blots

First, we analyzed whether cholesterol levels can regulate inflammasome induction. We used the SIM-A9 cell line, which has been previously reported to express macrophage/microglia-specific proteins, such as CD68 and AIF1/IBA1, and have complete responsiveness to exogenous inflammatory stimulation [30]. Cells were incubated with a water-soluble cholesterol complex (CHO:MCD) for 1 h. The treatment resulted in a fourfold increase in total cholesterol levels (31.8 ± 2.5 μg/mg protein vs. 6.9 ± 1.1 μg/mg protein; *P* = 0.0001, *n* = 3) (Fig. 1a), which was uniformly distributed within the cells as shown by filipin staining (Fig. 1b). To trigger inflammasome activation, cells were incubated with LPS (10 μg/ml) in combination with ATP (5 mM) or MDP (10 μg/ml), an immunoreactive peptide present in bacterial peptidoglycans and sensed by NLRP1 and NLRP3 receptors [37, 38]. In both cases, the expression levels of NLRP3 and the proforma of IL-1β were up-regulated, while the levels of NLRP1 remained unaltered (Fig. 1c). Furthermore, the rise of NLRP3 levels was significantly enhanced by cellular cholesterol enrichment (Fig. 1c). In agreement with the two-hit hypothesis proposed for NLRP3 inflammasome activation [8], no changes were observed in the levels of any of the inflammasome-related proteins when cells were incubated with an NLRP3 inflammasome inducer, such as MSU (150 μg/ml) [39], without priming with LPS (Fig. 1c). Next, we evaluated the inflammasome assembly using green fluorescent protein-tagged ASC (ASC-GFP). The conjugate, normally distributed homogeneously in the cytosol, is detected as condensed specks when associated with inflammasome receptors. As is shown, ASC oligomerization was observed only after LPS and MDP treatment (Fig. 1d). As a direct readout for inflammasome function, we also analyzed CASP1 activation in live cells using the FAM-YVAD-FMK (FAM-FLICA) probe that becomes covalently coupled to the active form of the enzyme and is retained within the cell. As expected, LPS plus MDP exposure resulted in an increased number of cells with dots of FAM-FLICA staining (Fig. 1e); however, the presence of FAM-FLICA-positive cells was not significantly enhanced after cholesterol enrichment (Fig. 1e), indicating that the endotoxin-mediated activation of the inflammasome pathway is independent of cholesterol levels in microglial cells. In response to CASP1 activation, cells incubated with PAMPs (LPS and MDP) released mature IL-1β to the culture media, quantified by ELISA (Fig. 1f). Unexpectedly, the levels of the cytokine were significantly lower in the supernatant of cholesterol-enriched cells (Fig. 1f). On the other hand, cell viability remained unchanged up to 16 h of inflammasome induction, although it was compromised later (Fig. 1g), indicating that the early release of IL-1β is not related to cell demise. Interestingly, previous studies showed that several cytokines, including IL-1β, can be secreted within EVs [40, 41], an event modulated upon cell activation [41]. Given that encapsulated cytokines are not detected by cell-free cytokine assays such as ELISA, we treated the supernatants with 1% Triton X-100 for 10 min. The detergent-treated media from cholesterol-enriched cells displayed significantly higher levels of IL-1β (Fig. 1f), indicating that high cholesterol load stimulates the release of EV-encapsulated IL-1β after inflammasome activation. EV-associated cytokines have been reported to be more stable and biologically active [41]. The functional significance of these cholesterol-mediated changes is unknown and would need further investigation.

### Activated microglia display a neuroprotective phenotype when intracellular cholesterol levels are increased

**Fig. 2:**
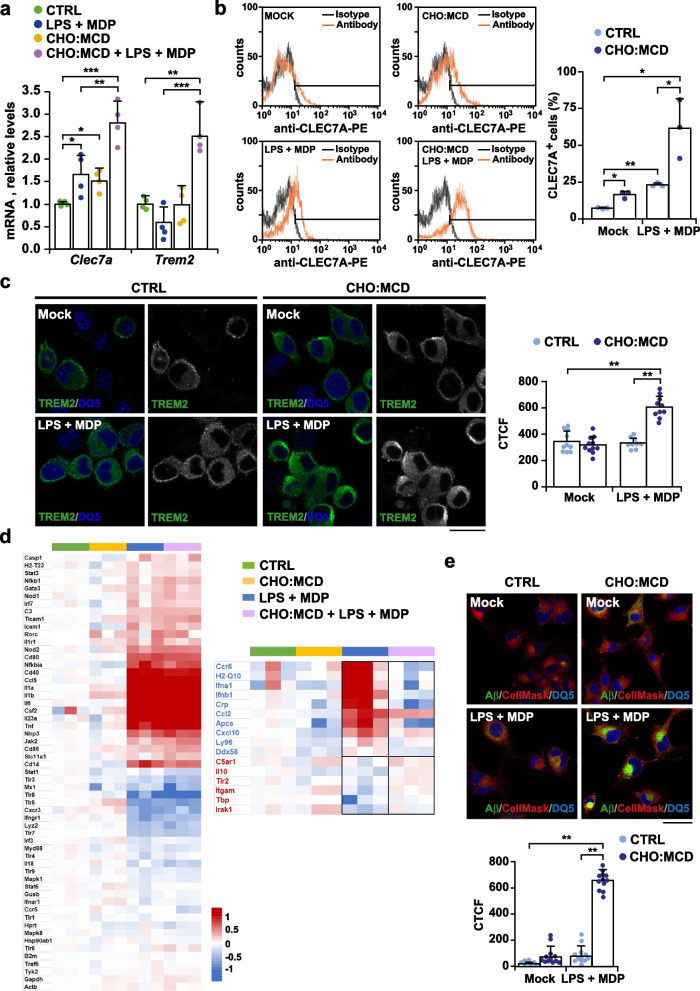
High cholesterol burden attenuates the pro-inflammatory phenotype displayed by PAMP-activated SIM-A9 microglial cells. Cells were cholesterol-enriched by incubation with the CHO:MCD complex for 1 h. After 4 h of recovery, cells were stimulated with LPS (10 μg/ml) plus MDP (10 μg/ml) for 16 h. **a** *Trem2* and *Clec7a* mRNA expression levels analyzed by selfie qRT-PCR. Transcript copies were normalized to total genomic DNA and reported as relative levels referred to the expression in CTRL cells (*n* = 4 independent experiments). **b** CLEC7A levels quantified by flow cytometry (*n* = 3). Histogram plots provide representative data from 3 independent experiments. **c** Representative confocal immunomicrographs from 3 independent experiments showing enhanced cell surface presence of TREM2 in cholesterol-enriched cells after PAMP exposure. Nuclei were stained with DRAQ5 (blue). Images from the green channel corresponding to TREM2 immunostaining are shown in black and white. Plot represents TREM2 levels per cell, quantified as the CTCF of the green channel (*n* = 9–10 non-overlapping images). Scale bar: 25 μm. **d** Heat map depicting transcriptional changes in gene expression assayed by qRT-PCR using an innate and adaptive immune response gene array. Each probe set is represented in a blue-red row Z-score scale with red indicating high expression and blue low expression. The panel on the right shows the genes whose endotoxin-mediated changes in expression are up/down-regulated by cholesterol enrichment (*n* = 3 independent experiments). See Additional file 2 for array dataset. **e** Immunofluorescence measurement of phagocytosis. Representative confocal images of microglia incubated with fluorescent Aβ and counterstained with CellMask (cytosol/plasma membrane, red) and DRAQ5 (nuclei, blue) (*n* = 3 independent experiments). Scale bar, 25 μm. Plot represents Aβ phagocytosed per cell, quantified as the CTCF of the green channel (*n* = 12 non-overlapping images). One-way ANOVA followed by the Tukey–Kramer test was applied to calculate statistical significance (*P ≤ 0.05, **P ≤ 0.01, ***P ≤ 0.001)

As previously mentioned, specific genetic signatures have been identified within the spectrum of microglial changes associated with neurodegeneration, referred to as DAM and MGnD phenotypes [4, 5]. Remarkably, two major regulators of these pathways of phenotypic transition are somehow related to cholesterol metabolism, including APOE, involved in cholesterol extracellular transport, and TREM2, which has been recently shown to regulate the intracellular movement of cholesterol in microglia [42]. Given this apparent relationship with cholesterol, we tested whether changes in intracellular cholesterol content may contribute to the transition of microglia to a DAM/MGnD phenotype. We analyzed the mRNA expression levels of *Trem2* and *Clec7a* (c-type lectin domain family 7, member a), a pattern recognition receptor related to phagocytosis [43], by selfie qRT-PCR. Results showed that cholesterol enrichment upregulated the expression of *Trem2* mRNA in microglia exposed to LPS and MDP (Fig. 2a). Regarding *Clec7a*, although both bacterial endotoxin exposure and high cholesterol load separately stimulated the transcription levels of the receptor, the increase of *Clec7a* mRNA levels in microglia cells was significantly enhanced when the LPS plus MDP treatment was combined with cholesterol enrichment (Fig. 2a). Furthermore, the transcriptional changes correlated with an enhanced cell surface availability of both CLEC7A and TREM2 receptors (Fig. 2b, c), thus confirming that the rise of cholesterol levels is sensed by microglia as a signal to engage a DAM/MGnD signature. To complement these results and gain a broader view of the regulatory role of cholesterol on the inflammatory transcriptomic profile of active microglia, we used a microarray with a large panel of different innate and adaptative immune-related genes (Fig. 2d). Consistent with an induced inflammatory response, we found that pro-inflammatory cytokines (*Il-1a, Il-1b, Il-6,* and *Tnf*) and related gene transcripts involved in pro-inflammatory signaling pathways (*Nfkb1, Nfkbia, Jak2,* and *Stat3*) were highly upregulated in cells exposed to LPS and MDP regardless of the cholesterol level (Fig. 2d). Also, as part of the anti-microbial response, several NOD-like receptor family members were stimulated, including *Nod1/2* and *Nlrp3*, corroborating inflammasome induction. Intriguingly, high intracellular cholesterol load in PAMP-activated microglia attenuated the expression of genes related to innate antiviral response (*Ifna2*, *Ifnb1*, *Ddx58*), genes involved in chemotaxis (*Ccr6*, *Cd14*, *Cxcl10*), and members of the pentraxin family, such as *Crp* (C-reactive protein) and *Apcs* (amyloid P component, serum). Conversely, among the genes upregulated after cholesterol enrichment, we found anti-inflammatory *Il-10*, *Itgam* that encodes CD11b, a component of the macrophage-1 antigen complex (also known as complement receptor 3) involved in phagocytosis, and the gene that encodes toll-like receptor 2, which has been described as a receptor for Aβ phagocytosis [44].

Given that the disease-associated cellular reprogramming has also been linked to increased phagocytosis [4, 5], we next studied if high cholesterol levels can modulate the phagocytic capacity of SIM-A9 cells. We incubated the cells with HiLyte Fluor 488-labeled Aβ for 4 h and then analyzed its uptake by confocal microscopy. Micrographs revealed a significant increase in the amount of fluorescent Aβ phagocytosed when cells were cholesterol-enriched before LPS and MDP exposure (Fig. 2e). Moreover, the enhanced phagocytosis was accompanied by a change of cell shape. In contrast to control cells that showed a smaller round cell body, cholesterol-enriched cells adopted a more irregular and amoeboid-like morphology resembling a microglial reactive-phenotype (Fig. 2e).

**Fig. 3:**
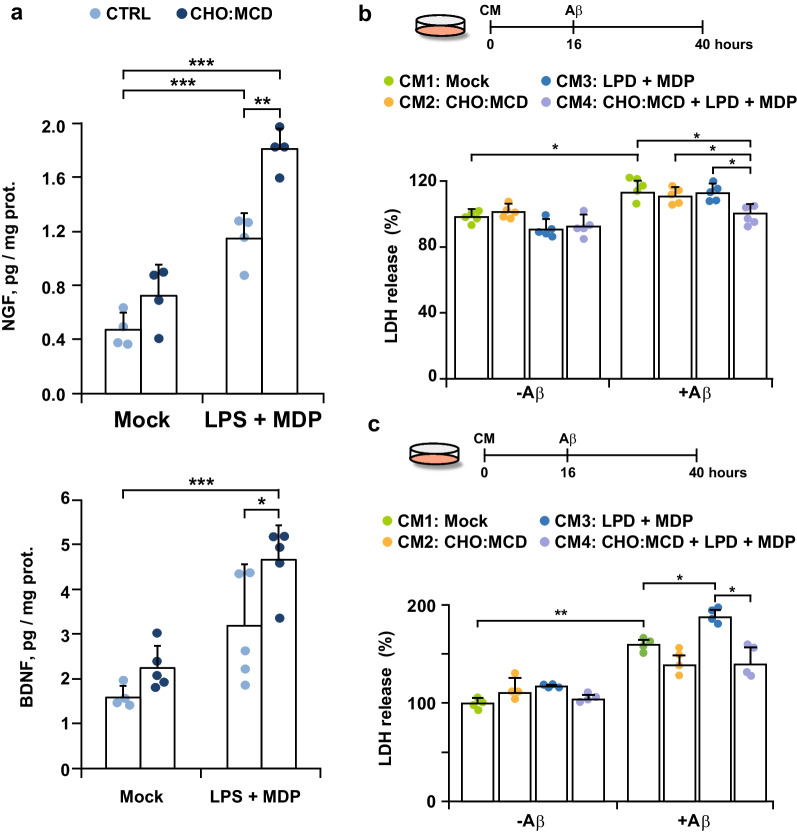
Neuroprotective effect of conditioned media from activated microglia after cholesterol enrichment. Cells were cholesterol-enriched by incubation with the CHO:MCD complex for 1 h. After 4 h of recovery, cells were stimulated with LPS (10 μg/ml) plus MDP (10 μg/ml) for 16 h. **a** NGF and BDNF secretion. Cell culture media were collected after the indicated treatments and the levels of both neurotrophins were analyzed by ELISA. The protein concentration of cell extracts was used for data normalization (*n* = 4–5 independent experiments). **b** and **c** Analysis of cell death by LDH assay. Primary neuronal cell cultures (**b**) and SH-SY5Y cells (**c**) were first incubated for 16 h with conditioned media (CM) from SIM-A9 cells treated as indicated (CM1: mock, CM2: CHO:MCD exposure for 1 h, CM2: 10 μg/ml LPS + 10 μg/ml MDP induction for 16 h, and CM4: CHO:MCD exposure for 1 h followed by LPS + MDP induction for 16 h). Then, cells were exposed to Aβ for 24 h. LDH activity was determined in the cell culture media and normalized to total cellular LDH content. Results are expressed as % relative to the untreated control values. (*n* = 4–5 independent experiments). One-way ANOVA followed by the Tukey–Kramer test was applied to calculate statistical significance (*P ≤ 0.05, **P ≤ 0.01, ***P ≤ 0.001)

Among the protective features of activated microglia, there is reported to be stimulated expression and release of neurotrophic factors [45, 46], which have beneficial effects on the survival and functionality of the microglia themselves and surrounding neuronal cells [47]. Thus, we next analyzed if the rise of cholesterol levels in PAMP-activated microglia stimulates the secretion of neurotrophic factors. We collected the culture media after LPS and MDP treatment and determined the levels of different neurotrophin family members, including nerve growth factor (NGF), brain-derived neurotrophic factor (BDNF), neurotrophin 3 (NT3), and neurotrophin 4 (NT4). Whereas NT3 and NT4 were undetectable in all of the supernatants analyzed (data not shown), conditioned media from PAMP-activated cells showed increased levels of NGF and BDNF (although in the latter case, differences did not reach statistical significance at a α level of 0.05) (Fig. 3a). The secretion of both neurotrophins was further stimulated after cellular cholesterol enrichment (Fig. 3a). In parallel, we tested the protective effect of these conditioned media on neuronal cells exposed to Aβ. Embryonic cortical/hippocampal neurons isolated from WT mice were first incubated with 24 h-conditioned media from PAMP-stimulated SIM-A9 cells (with and without cholesterol enrichment) for 16 h before being treated with Aβ (5 μM) for 24 h. We observed a significant reduction of Aβ cytotoxicity when we used conditioned media from cholesterol-enriched cells treated with the inflammasome inducers (Fig. 3b). We also analyzed the effect of microglia conditioned media against Aβ insult in SH-SY5Y neuroblastoma cells (Fig. 3c). In this case, conditioned media from PAMP-stimulated SIM-A9 cells exacerbated Aβ-induced cell death. The cytotoxicity elicited by the peptide was markedly lower when the SIM-A9 cells were cholesterol-enriched prior to LPS- and MDP-mediated activation (Fig. 3c), thus reproducing in part the cholesterol-protective effect observed in primary neuronal cultures. Overall, these findings suggest that the rise of cellular cholesterol content favors the transition of microglia to a protective phenotype after PAMP exposure, stimulating phagocytosis and the release of neurotrophic factors via an inflammasome-dependent mechanism.

### Cholesterol regulates pathogen-mediated inflammasome activation in SH-SY5Y cells resulting in enhanced death

**Fig. 4:**
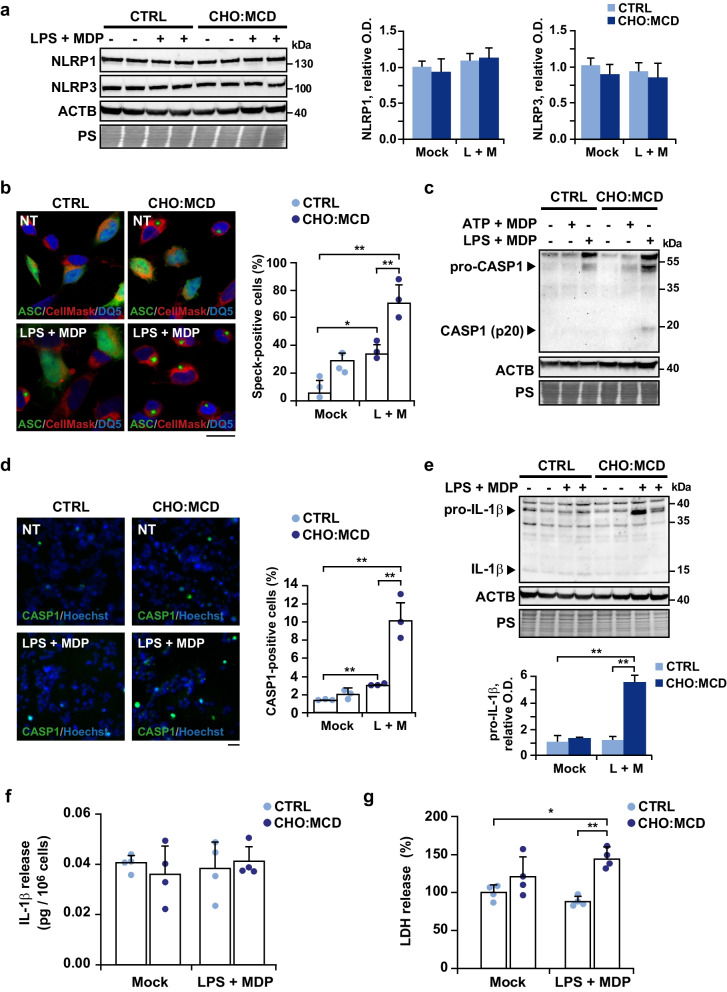
Cholesterol-enriched neuroblastoma SH-SY5Y cells show enhanced endotoxin-mediated activation of NLRP3 inflammasome. After treatment with the CHO:MCD complex cells were co-incubated with LPS and MDP (L + M, 10 μg/ml each for 16 h) or MDP (10 μg/ml, 16 h) plus ATP (5 mM, 1 h).** a** Western blot analysis of NLRP1 and NLRP3 in cellular extracts. (*n* = 3 independent experiments). **b** Representative confocal images of oligomeric ASC. Cells were transfected with a plasmid encoding an ASC:GFP fusion protein and 48 h later were treated as indicated. Cells were counterstained with CellMask (cytosol/plasma membrane, red) and DRAQ5 (nuclei, blue). Scale bar: 25 μm. Data in the graph are expressed as % of speck-positive cells of total transfected cells (*n* = 3 independent experiments). **c** Representative immunoblots from 3 independent experiments showing pro- and cleaved CASP1 (active product of 20 kDa) in cellular extracts. **d** Representative micrographs of CASP1-positive cells (green). The fluorescent CASP1 inhibitor was added during the inflammasome induction period (16 h). Nuclei were stained with Hoechst 33342 (1 μg/ml). Scale bar: 100 μm. Data in the graph are expressed as % of CASP1-positive cells to the total Hoechst-stained cells (blue) (*n* = 3 independent experiments). **e** Representative immunoblots of pro- and mature IL-1β in cellular extracts. (*n* = 4 independent experiments). **f** Levels of IL-1β in the cell culture supernatants. Values were normalized to the total protein content and expressed as % relative to the untreated control values (*n* = 4 independent experiments). **g** Cell death by the LDH assay. Results are expressed as % to the untreated control values (*n* = 4 independent experiments). In western blots, ACTB/actin β and ponceau S (PS) staining were used as loading controls and optical density (O.D.) values of the bands representing the specific protein immunoreactivity were normalized to PS staining. One-way ANOVA followed by the Tukey–Kramer test was applied to calculate statistical significance (*P ≤ 0.05, **P ≤ 0.01). See Additional file 1: Fig. S6 for uncropped blots

In addition to microglia, non-glial cells such as neurons participate in innate immunity. Accordingly, previous studies have shown inflammasome activation in neurons exposed to different cellular stressors, including Aβ [48–50] and we wanted to see if cholesterol may also play a regulatory role as observed in microglia. We used cholesterol-enriched SH-SY5Y neuroblastoma cells, which we had previously characterized and shown the link between high cholesterol, reduced mitochondrial GSH levels, and greater susceptibility to the toxic Aβ peptides [20, 51]. The incubation with the CHO:MCD complex for 1 h resulted in a threefold increase of total cholesterol levels (32 ± 1.7 μg/mg protein vs. 10 ± 2.5 μg/mg protein; *P* = 0.0001, *n* = 4–7) accompanied by a marked depletion of mitochondrial GSH levels (Additional file 1: Fig. S2), in contrast to SIM-9 cells in which cholesterol enrichment did not modify mitochondrial GSH content (1.96 ± 1.18 nmol/mg protein vs. 2.19 ± 0.71 nmol/mg protein; *P* = 0.78, *n* = 3). After PAMP treatment, the expression levels of NLRP1 and NLRP3 remained unchanged regardless of intracellular cholesterol levels (Fig. 4a). In parallel, we analyzed the inflammasome assembly by ASC speck visualization, and found that the incubation of the cells with LPS and MDP induced ASC oligomerization (Fig. 4b). Similarly, the increase in cholesterol load alone also seemed to promote the appearance of ASC specks (although in that case, changes did not reach statistical significance at an α level of 0.05). However, the number of speck-positive cells was markedly increased when PAMP-activated cells were subjected to cholesterol enrichment (Fig. 4b), accompanied by enhanced expression of pro-CASP1 and self-cleavage, as revealed by the presence of the active 20-kDa fragment in the immunoblots (Fig. 4c). Previous studies showed that inflammasome activation by MDP requires ATP [37]; however, in our conditions, cells incubated with MDP and ATP did not show enhanced CASP1 cleavage (Fig. 4c). CASP1 activation was also assessed using the FAM-FLICA probe (Fig. 4d). The higher sensitivity of this method compared to western blot analysis allows the identification of active caspase in cells simply exposed to the bacterial toxins. Again, we found a greater number of CASP1-positive cells after modifying intracellular cholesterol levels, reflecting enhanced inflammasome activation (Fig. 4d). The proforma of IL-1β was also upregulated in cell lysates of cholesterol-enriched cells after PAMP-mediated inflammasome induction (Fig. 4e); however, despite the presence of active CASP1, we did not find enhanced levels of its mature form neither in the cell lysates (Fig. 4e) nor in the supernatants, as analyzed by ELISA (Fig. 4f). Instead, unlike in microglia, the viability of cholesterol-enriched neuronal cells, assessed by LDH assay (Fig. 4g) and trypan blue exclusion (see Additional File 1: Fig. S3), were compromised after LPS and MDP exposure, suggesting the engagement of the pyroptotic pathway in neuronal cells.

### Aβ-induced inflammasome assembly in neuronal cells is enhanced by cholesterol-mediated depletion of mitochondrial GSH content

**Fig. 5:**
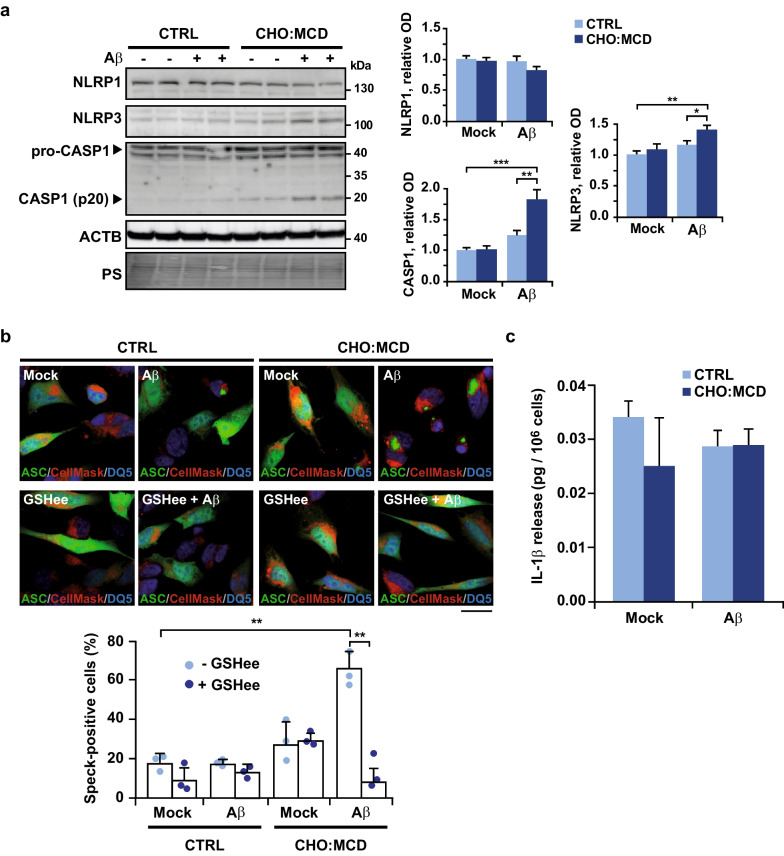
Cholesterol overload promotes inflammasome activation in SH-SY5Y cells in response to Aβ exposure. For cholesterol enrichment, cells were treated with the CHO:MCD complex for 1 h. After 4 h of recovery, cells were exposed to Aβ (10 μM) for 24 h. In some cases, cells were pre-treated with GSH ethyl ester (GSHee, 4 mM) for 30 min before Aβ treatment. **a** Western blot analysis of NLRP1, NLRP3 and pro- and cleaved CASP1 (self-cleavage and active product of 20 kDa) in cellular extracts. The ACTB/actin β immunoblot and ponceau S (PS) staining were used as loading controls. Optical density (O.D.) values of the bands representing the specific protein immunoreactivity were normalized to the values of the corresponding PS staining (*n* = 4 independent experiments). **b** Representative confocal images of oligomeric ASC forms. To monitor ASC-dependent inflammasome assembly cells were transfected with a plasmid encoding an ASC:GFP fusion protein and counterstained with CellMask (cytosol/plasma membrane, red) and DRAQ5 (nuclei, blue). Speck formation (seen as an aggregate) was determined by confocal microscopy and the number of speck-positive cells of total transfected cells was quantified (*n* = 3 independent experiments). Scale bar, 25 μm.** c** Levels of IL-1β in the cell culture supernatants after Aβ incubation (*n* = 4 independent experiments). One-way ANOVA followed by the Tukey–Kramer test was applied to calculate statistical significance (*P ≤ 0.05, **P ≤ 0.01, ***P ≤ 0.001). See Additional file 1: Fig. S6 for uncropped blots

Aβ has been identified as an inducer of inflammasomes in both microglia and neuronal cells [12, 48–50, 52]. Nonetheless, although different mechanisms of activation have been proposed, including Aβ-induced mitochondrial ROS generation [16], the process still needs to be fully characterized in neurons. We found up-regulated expression of NLRP3 in the cholesterol-enriched cells after 24-h incubation with Aβ (10 μM) (Fig. 5a), whereas NLRP1 levels remained unchanged (Fig. 5a). Moreover, consistent with an enhanced inflammasome assembly, confocal microscopy analyses revealed a significantly increased number of ASC speck-positive cells when cellular cholesterol was enriched before Aβ exposure (Fig. 5b). Accordingly, immunoblots of total cellular lysates showed stimulated CASP1 self-cleavage and release of the active 20 kDa fragment (Fig. 5a). However, the enhanced inflammasome activation in CHO:MCD-treated cells did not lead to a greater release of mature IL-1β into the culture media (Fig. 5c), thus reproducing the same outcomes obtained when LPS and MDP were used as inflammasome inducers. Interestingly, treatment with GSHee, a cell membrane-permeable derivative of GSH that restored the depleted pool of mitochondrial GSH in cholesterol-enriched SH-SY5Y cells (Additional file 1: Fig. S2), markedly reduced the presence of ASC speck–positive cells after Aβ exposure (Fig. 5b). Altogether, our findings indicate that high intracellular cholesterol content in neuronal cells promotes Aβ-induced inflammasome activation by compromising the mitochondrial antioxidant defense.

### Cholesterol overload in neuronal cells promotes pyroptosis by enhancing Aβ-induced mitochondrial oxidative stress

**Fig. 6:**
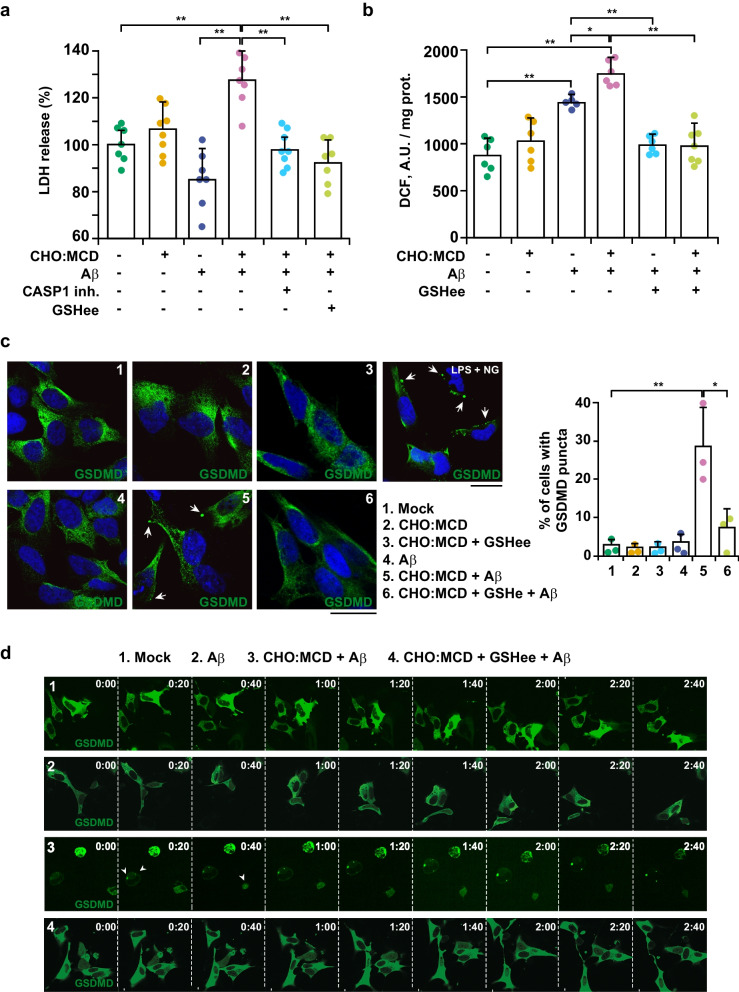
Cholesterol-enriched SH-SY5Y cells display increased susceptibility to Aβ-induced pyroptosis, which is prevented by GSHee treatment. After treatment with the CHO:MCD complex for 1 h, cells were exposed to Aβ (10 μM) for 24 h. In some cases, cells were pre-treated with GSH ethyl ester (GSHee, 4 mM) or a cell-permeable CASP1 inhibitor (10 μM) for 30 min before Aβ treatment. **a** Analysis of cell death by the LDH assay. LDH activity was determined in the cell culture media and normalized to total cellular LDH content. Results are expressed as % of untreated control values. (*n* = 6 independent experiments). **b** Intracellular ROS generation assessed by DCF fluorescence intensity (A.U.: arbitrary units). (*n* = 6–8 independent experiments). **c** Representative confocal immunomicrographs showing apical GSDMD puncta (white arrows) in cholesterol-enriched cells after Aβ exposure. Nuclei were stained with DRAQ5 (blue). Cells incubated with LPS (100 ng/ml) for 6 h followed by nigericin (NG, 10 μM) for 2 h were used as positive controls. Scale bar: 15 μm. Data in the graph are expressed as % of cells with GSDMD puncta over total cells. (*n* = 3 independent experiments). **d** Time-lapse microscopy of cells expressing mNeoGreen-GSDMD. Image series (20-min frames) depict the 2 h leading up to the loss of membrane integrity and cell round-up characteristic of necroptotic death in cholesterol-enriched cells exposed to Aβ. Presence of GSDMD puncta is indicated by white arrows. See Additional file 3, Additional file 4, Additional file 5 and Additional file 6 for the corresponding movies. One-way ANOVA followed by the Tukey–Kramer test was applied to calculate statistical significance (*P ≤ 0.05, **P ≤ 0.01)

Previous studies have linked the pathway of inflammasome signaling evoked by Aβ in neurons to pyroptosis [48, 49], a programmed cell death that features cell swelling and plasma membrane disruption. To evaluate pyroptosis in our cell cultures, we first quantified the release of LDH to the culture medium, as readout of membrane permeabilization. The CHO:MCD-treated SH-SY5Y cells exposed to Aβ displayed enhanced LDH release, which was partially counteracted by the incubation with a cell-permeable CASP1 inhibitor (Fig. 6a). Remarkably, a comparable degree of protection against Aβ-induced cytotoxicity was achieved by pre-incubating the cells with GSHee (Fig. 6a), which reverted the ROS generation to control levels (Fig. 6b). Recent studies have identified the pore-forming protein GSDMD as the necessary executor of pyroptosis [10]. Once cleaved by CASP1 or LPS-activated caspase 11 (caspase 4/5 in humans), the amino-terminal domain of GSDMD is liberated and can be integrated into the cell membrane where it oligomerizes to form large pores, which ultimately result in cell swelling and membrane rupture. To confirm the involvement of GSDMD in the cell death exhibited by cholesterol-enriched SH-SY5Y cells exposed to Aβ, we assessed the intracellular distribution of GSDMD by immunolabeling and confocal microscopy analysis (Fig. 6c). Results showed that GSDMD was homogenously distributed in the cytosol of the control cells after the Aβ insult. In contrast, in cholesterol-enriched cells, the exposure to the toxic peptide led to the appearance of GSDMD puncta near the cell limits (Fig. 6c), and this was prevented by GSHee pre-treatment (Fig. 6c). Similar punctated structures of the pyroptotic marker were observed in LPS-primed cells incubated with the canonical NLRP3 inflammasome activator nigericin (Fig. 6c). Additionally, using time-lapse confocal microscopy, we performed a live visualization of GSDMD-driven pyroptotic death in individual cells (Fig. 6d, see movies in Additional file 3, Additional file 4, Additional file 5 and Additional file 6). We confirmed the presence of GSDMD puncta in cholesterol-enriched cells exposed to Aβ that was observed concurrently as the cell swelled and died (Fig. 6d, see movie in Additional file 5). Conversely, the pyroptotic mediator showed a diffused presence in the cytosol, and the cytotoxic effect of Aβ was abolished when cholesterol-loaded cells were treated with GSHee (Fig. 6d, see movie in Additional file 6). Therefore, our results indicate that cholesterol levels regulate the viability of neuronal cells exposed to Aβ, inducing pyroptosis, a proinflammatory death, which is counteracted by the recovery of mitochondrial GSH levels and the consequent reduction of Aβ-induced oxidative stress.

### Conditioned media from cholesterol-enriched neuronal cells exposed to Aβ impair microglia phagocytosis

**Fig. 7:**
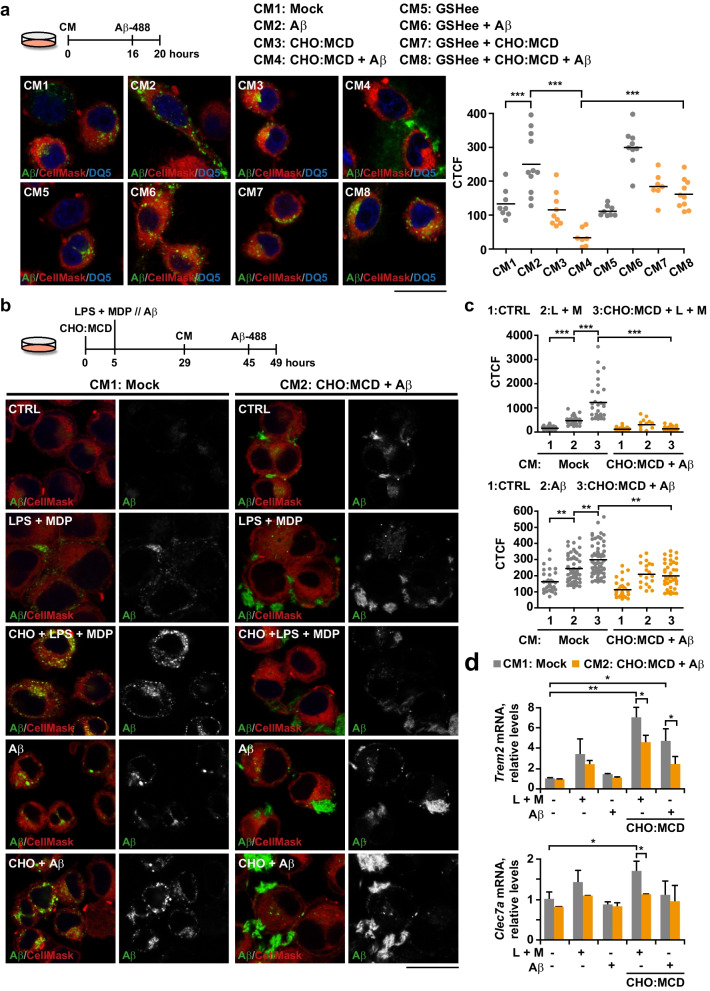
Conditioned media from cholesterol-enriched SH-SY5Y cells exposed to Aβ alter the phagocytic capacity of microglia. Conditioned media were obtained from SH-SY5Y cells cholesterol-enriched (CHO:MCD complex, 1 h) and then treated with Aβ (10 μM, 24 h). In some cases, cells were pre-treated with GSH ethyl ester (GSHee, 4 mM) for 30 min before Aβ exposure. SIM-A9 cells were incubated with the conditioned media for 16 h and then HiLyte Fluor 488-labeled Aβ (1 μM) was added for 4 h. **a** and **b** Representative confocal micrographs from 3 independent experiments showing Aβ phagocytosis (green). In **b**, SIM-A9 cells were cholesterol-enriched with the CHO:MCD complex for 1 h and after 4-h recovery were primed with LPS (10 μg/ml) plus MDP (10 μg/ml) (L + M) for 16 h or Aβ (10 μM) for 24 h before exposure to conditioned media from SH-SY5Y cells. Cells were counterstained with CellMask (cytosol/plasma membrane, red) and DRAQ5 (nuclei, blue). Images from the green channel corresponding to fluorescent-labeled Aβ are shown in black and white. Scale bars: 15 μm. **a** and **c** Plots represent Aβ phagocytosed per cell, quantified as the corrected total cell fluorescence (CTCF) of the green channel (*n* = 9–12 non-overlapping images). **d** *Trem2* and *Clec7a* mRNA expression levels analyzed by selfie qRT-PCR. Transcript copies were normalized to total genomic DNA and reported as relative levels referred to the expression in mock cells. (*n* = 4 independent experiments). One-way ANOVA followed by the Tukey–Kramer test was applied to calculate statistical significance (*P ≤ 0.05, **P ≤ 0.01, ***P ≤ 0.001)

**Fig. 8:**
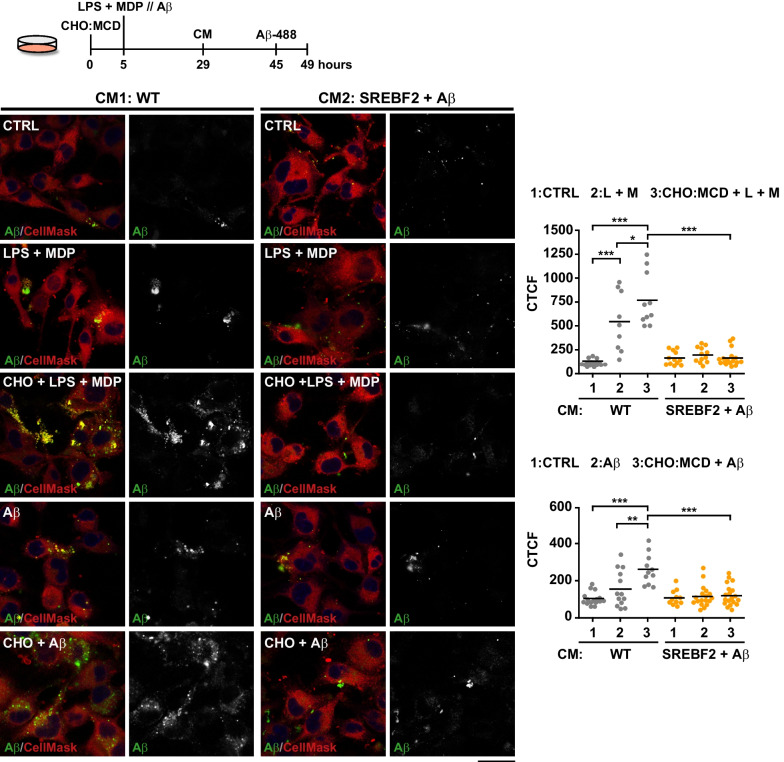
Conditioned media from Aβ-treated SREBF2 neurons inhibit phagocytosis in activated and cholesterol-enrichment microglia. Conditioned media were obtained from WT and SREBF2 primary neuronal cultures treated with Aβ (1 μM, 48 h). SIM-A9 cells were cholesterol-enriched with the CHO:MCD complex for 1 h and after 4-h recovery were primed with LPS (10 μg/ml) plus MDP (10 μg/ml) (L + M) for 16 h or Aβ (10 μM) for 24 h before exposure to conditioned media for 16 h. HiLyte Fluor 488-labeled Aβ (1 μM) was added for 4 h to evaluate phagocytosis by confocal microcopy. Shown are representative confocal micrographs from 2 independent experiments. Cells were counterstained with CellMask (cytosol/plasma membrane, red). Images from the green channel corresponding to fluorescent-labeled Aβ are shown in black and white. Scale bars: 15 μm. Plots represent Aβ phagocytosed per cell, quantified as the corrected total cell fluorescence (CTCF) of the green channel (*n* = 5 non-overlapping images). One-way ANOVA followed by the Tukey–Kramer test was applied to calculate statistical significance (*P ≤ 0.05, **P ≤ 0.01, ***P ≤ 0.001)

Finally, we evaluated whether cholesterol-promoted pyroptosis in neuronal cells after Aβ exposure can affect microglial behavior. We incubated SIM-A9 microglia with conditioned media of Aβ-treated SH-SY5Y cells with and without cholesterol enrichment and analyzed their phagocytic capacity by confocal microscopy using HiLyte Fluor 488-labeled Aβ. Results showed that Aβ uptake was significantly increased in the microglia exposed to conditioned media of SH-SY5Y cells treated with Aβ (Fig. 7a), which can be attributable to a stimulation of the phagocytic response by the unlabeled fibrillar Aβ present in the conditioned media, as described previously [53]. In contrast, phagocytosis was severely affected in the presence of conditioned media from cholesterol-enriched SH-SY5Y cells exposed to Aβ (Fig. 7a). Interestingly, the inhibitory effect of these media was abolished with GSHee pre-treatment (Fig. 7a), which prevented neuronal pyroptosis (Fig. 6d). A similar reduction of Aβ-engulfment capacity was observed in activated microglia, primed with LPS and MDP or Aβ for 24 h before being subjected to conditioned media of pyroptotic cells (Fig. 7b, c). Furthermore, in these cells, impaired phagocytosis was accompanied by a significant decrease of *Trem2* and *Clec7a* mRNA expression, both of which were upregulated when intracellular cholesterol content was enriched before PAMP or Aβ incubation (Fig. 7d). Cross-talk analyses between neurons and microglia were validated using mouse primary neuronal cultures, thereby keeping species equality with SIM-A9 cells. Embryonic cortical-hippocampal neurons were isolated from WT and transgenic mice that overexpress the cholesterol-related transcription factor SREBF2. In previous studies, we showed that SREBF2 neuronal cultures display high intracellular cholesterol levels (total and mitochondrial) that correlate with an increased susceptibility to Aβ [20]. Neuronal cultures at DIV7 were exposed to Aβ (1 μM), which induced expression of inflammasome-related proteins and CASP1 activity in SREBF2 cells (Additional file 1: Fig. S4). The 48-h conditioned media were then supplied to SIM-A9 microglia, previously activated with LPS and MDP or Aβ for 24 h. Phagocytosis was tested by confocal microscopy using the fluorescent Aβ peptide (Fig. 8). Results showed that conditioned media from SREBF2 neuronal cultures incubated with Aβ inhibited the enhanced phagocytosis displayed by activated and cholesterol-enriched microglia. In sum, our results suggest that microglia sense the damage signals released by pyroptotic neurons, inhibiting TREM2-mediated signaling pathways that trigger phagocytosis. Thus, cholesterol overload in neurons is an emerging key regulatory factor in the microglia-neuron relationship, which, by modulating microglial phagocytosis, can increase Aβ deposition and contribute to the perpetuation of inflammation in AD.

## Discussion

In the present study, we show that changes in the cellular cholesterol content regulate the inflammasome pathway induced by classical stimuli like pathogen-associated molecules and Aβ peptide. However, while in microglia, inflammasome induction under high cholesterol levels leads to a neuroprotective phenotype with upregulated phagocytosis, in SH-SY5Y neuronal cells the activation of the inflammasome increases pyroptotic death and is linked to enhanced mitochondrial oxidative stress.

Pyroptotic markers have been described in AD mouse models and primary neuronal cultures exposed to Aβ [48, 49, 54]. Nonetheless, the underlying mechanism that drives neurons towards this fatal outcome once the inflammasome is activated is still unknown. We have previously shown that high mitochondrial cholesterol levels impair mitochondrial GSH transport in neuronal cells, reducing the antioxidant buffering against Aβ-induced mitochondrial ROS [20]. Consistent with these data, we observed that GSHee treatment, which restores the mitochondrial GSH content in cholesterol-enriched cells [25], significantly decreased ROS levels. Remarkably, the antioxidant effect of GSHee prevented inflammasome assembly and reduced cell death, thus pointing to mitochondrial oxidative stress as the trigger of the Aβ-induced pyroptosis observed in cholesterol-enriched neuronal cells. Corroborating the regulatory role of the GSH antioxidant system in NLRP3 inflammasome activation, recent studies have shown that the assembly of the complex requires the deglutathionylation of ASC by GSH transferase omega 1, under the control of mitochondrial ROS [55].

We found increased ASC oligomerization in cholesterol-enriched neuronal cells after PAMP and Aβ exposure. The presence of these ring-like perinuclear complexes is commonly used as an indicator of canonical inflammasome activation. Nonetheless, recent studies have also reported an inflammasome-independent ASC assembly (called pyroptosome) in response to potassium depletion, which can recruit and activate CASP1 and induce pyroptosis [56]. Therefore, the participation of pyroptosomes cannot be ruled out in cells exposed to toll-like receptor agonists (like LPS) with potassium-depleting agents such as ATP, nigericin, or MSU.

Pyroptotic executioner GSDMD is cleaved by CASP1 and the resultant GSDMD-N terminal fragment then selectively interacts with membrane lipids to form transmembrane pores [9, 10]. Consistent with pyroptosis induction, our analyses by confocal microscopy revealed the presence of GSDMD aggregates in close vicinity to the plasma membrane in cholesterol-enriched cells exposed to Aβ, which ultimately resulted in cell death as shown by time-lapse imaging. Moreover, we showed that GSHee pre-treatment prevented GSDMD puncta and Aβ-induced death, in line with recent studies that describe the participation of mitochondrial ROS in pyroptosis induction through GSDMD oxidation and promotion of CASP1-mediated cleavage [57]. Intriguingly, in clear opposition to our results, in vitro studies directed to elucidate the mechanics of GSDMD pore formation have suggested that cholesterol may exert an inhibitory effect [58]. These studies show that the addition of cholesterol to the lipid mixture in liposomes hinders the binding of the GSDMD N-terminal fragment to the lipid membrane [59]. However, the inhibitory effect disappears when liposomes are made from total lipid extract of both bacterial and eukaryotic sources, despite similar cholesterol content [59], indicating that this particular behavior would be restricted to a specific lipid mix.

In microglia, we found upregulated expression of inflammasome-related proteins and increased CASP1 activity elicited by PAMPs, which were accompanied by enhanced phagocytosis when cells were cholesterol-enriched. The transcriptional analysis showed upregulated gene expression of pro-inflammatory cytokines and related signaling pathways in PAMP-activated microglia regardless of cholesterol levels. Even though the transcriptional changes associated with high cholesterol were quite moderated, one thing that is worth noting is the attenuated expression of antiviral and interferon response genes, recently linked to microglia phenotypes that arise at late stages of neurodegeneration [2], and the induction of genes related to phagocytosis. In line with our findings, loss of the intracellular cholesterol carrier Niemann-Pick C1 (NPC1), which results in abnormal late endosomal/lysosomal lipid storage, has been shown to enhance the phagocytic capacity of microglia, increasing the uptake of myelin debris and Aβ [60]. Moreover, these functional changes are associated with a proteomic profile resembling the DAM signature and, more importantly, treatment with the cholesterol-lowering drug methyl-β-cyclodextrin rescues the microglial homeostatic signature in NPC1 disease models [60], which further supports the key role of cholesterol as a regulator of phenotypic changes in microglia associated with neurodegenerative processes. Intriguingly, recent RNA-seq analyses have revealed a unique transcriptomic signature in human AD microglia that differs from the DAM/MGnD profiles identified in AD mice [61]. However, despite dissimilarities, lipid-associated genes such as *APOE* and *LSR* (lipolysis-stimulated lipoprotein receptor) are upregulated in both human disease and mouse models [61]. Our data from real-time qPCR disclosed upregulated mRNA levels of *Clec7a* and *Trem2* in cholesterol-enriched microglia exposed to both pathogen-associated molecules and Aβ peptides, likely mirroring a microglial switch to damage/neurodegeneration-associated genetic signatures. CLEC7A is activated by β-1,3 glucans and regulates a range of cellular responses including phagocytosis, respiratory burst, and enhanced production of cytokines [43]. In turn, TREM2 has been described to sense a wide range of lipids associated with fibrillary Aβ and exposed during neuronal degeneration [62]. Of note, accumulation of cholesterol has also been reported in Aβ plaques [63], although it remains to be analyzed if the TREM2 receptor can recognize this pool of cholesterol. Moreover, the confocal microscopy analysis revealed increased TREM2 in PAMP-activated microglia after cholesterol enrichment, which seemed to accumulate heterogeneously in clusters at the cell surface. Interestingly, recent studies have shown that TREM2 shedding reduction and increased cell-surface-receptor load correlate with enhanced microglial survival and phagocytic activity; however, they become deleterious when sustained under pathological conditions such as AD [64].

Significantly, in addition to promoting phagocytosis, TREM2 has been shown to support microgliosis, a process of microglial expansion and clustering around Aβ plaques, by sustaining microglial survival [62]. In this regard, we found that conditioned media from cholesterol-enriched microglial cells exerted a protective effect in neuronal cells exposed to Aβ, which correlated with increased levels of BDNF and NGF, neurotrophic factors with well-known beneficial effects on neuronal survival and plasticity [65, 66]. Remarkably, recent studies have shown that genetic deletion of BDNF from microglia not only affects neuronal viability but can also interfere with self-renewal/proliferation of the microglia themselves [67]. Thus, further studies are warranted to evaluate the autocrine effect of the cholesterol-induced release of neurotrophins and the involvement of TREM2 in these events.

Finally, we found that conditioned media of both CHO:MCD-treated SH-SY5Y cells and primary neuronal cells overexpressing SREBF2 exposed to Aβ insult, completely abolished the enhanced phagocytic capacity of cholesterol-enriched microglia, which was likely due to the endogenous “danger” signals secreted or released after the Aβ insult. Our findings indicate that the microglia-neuron communication can ultimately modify microglia behavior, which is in line with growing evidence suggesting that the loss of microglial protection (dystrophic microglia), as opposed to reactive microglial, is the trigger for the cascade of events that lead to neuroinflammation and neurodegeneration in the early stages of AD [68].

## Conclusion

**Fig. 9:**
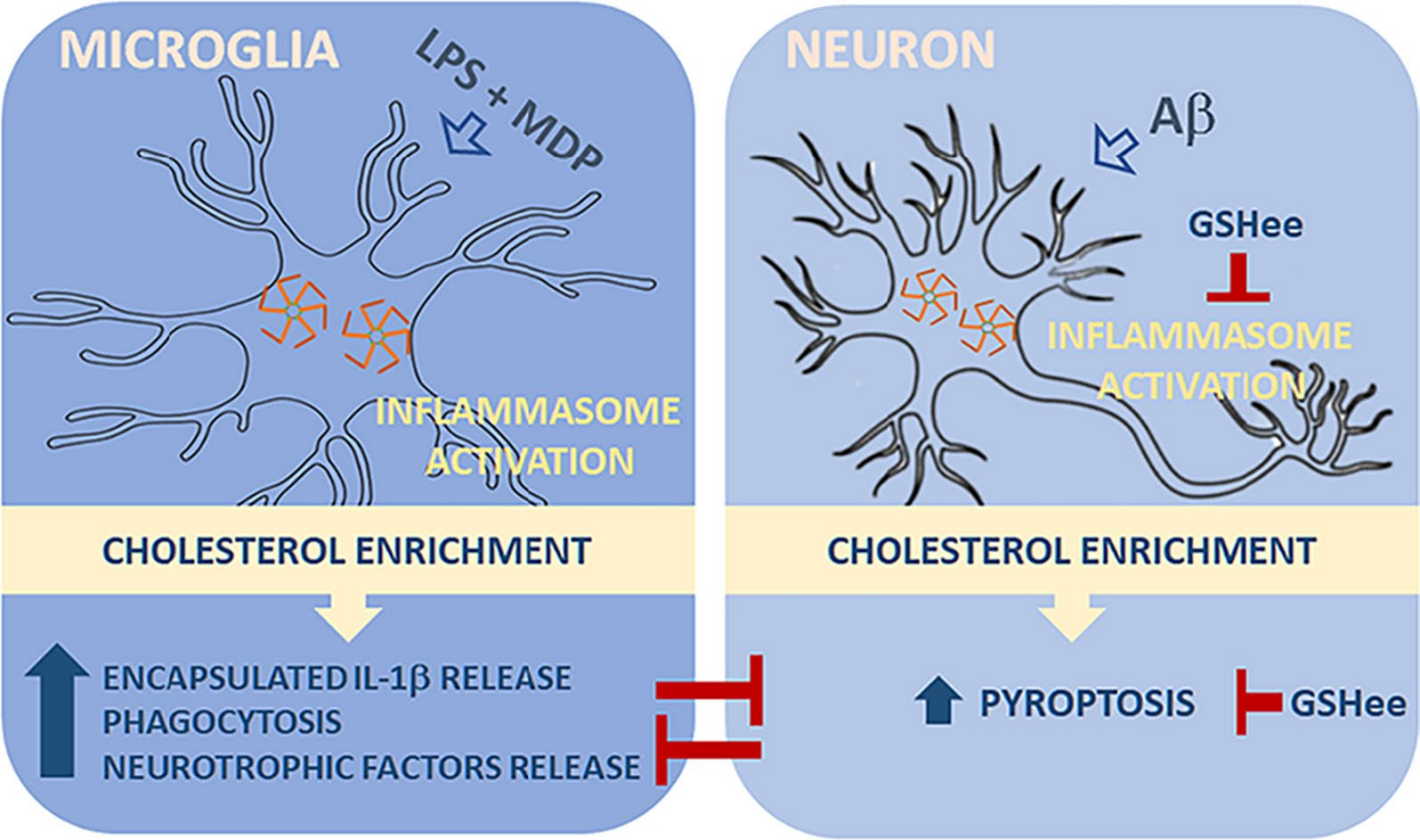
Proposed model illustrating the cholesterol regulation of the inflammasome-mediated immune response in microglia and neuronal cells

Based on our findings, we propose a model in which cholesterol levels act as a checkpoint of the immune response in AD, regulating the signaling pathways driven by inflammasome differentially in microglia and neurons (Fig. 9), thus, favoring a neuroprotective phenotype in microglia ultimately challenged by a pro-inflammatory neuronal death when intracellular cholesterol content rises and mitochondrial GSH is depleted. From a therapeutic point of view, approaches aimed at normalizing intracellular cholesterol levels would require selective adjustments to ensure that protective signals are not compromised in microglia, as well as the choice of appropriate timing to ensure maximum efficiency, considering that both mitochondrial cholesterol and oxidative stress accumulate over the disease progression. Once these issues are addressed, our findings indicate that the combination of immunomodulatory agents with cholesterol-lowering compounds or mitochondria-targeted antioxidants may be a strategy to be considered in AD treatment.

### Supplementary Information

**Additional file 1**. Fig. S1 EM of Aβ oligomers. Fig. S2 Mitochondrial GSH levels in SH-SY5Y cells. Fig. S3 Cell viability of SH-SY5Y cells after bacterial endotoxin49exposure. Fig. S4 Primary cortical neurons from SREBF2 mice show enhanced expression of inflammasome-related proteins and CASP-1 activation after Aβ exposure for 24h. Fig. S5 Uncropped scans of western blots included in Fig. 1. Fig. S6 Uncropped scans of western blots included in Fig. 4 and Fig. 5.

**Additional file 2**. SIM-A9 innate and adaptive immune response gene array dataset.

**Additional file 3**. Representative movie of mock-treated cells. Related to Figure 6d.

**Additional file 4**. Representative movie of cells incubated with Aβ (10 μM, 24 h). Related to Figure 6d.

**Additional file 5**. Representative movie of cholesterol-enriched cells incubated with Aβ (10 μM, 24 h). Related to Figure 6d.

**Additional file 6**. Representative movie of cholesterol-enriched cells pre-treated with GSH ethyl ester (GSHee, 4 mM, 30 min) before Aβ exposure. Related to Figure 6d.

## Data Availability

The datasets used and analyzed during the current study are available from the corresponding author on reasonable request.

## Abbreviations

AD: Alzheimer’s disease
APOE: Apolipoprotein E
ASC: Apoptosis-associated speck-like protein containing a CARD
BDNF: Brain-derived neurotrophic factor
CASP1: Caspase 1
CLEC7A: C-type lectin domain containing 7A
CTCF: Corrected total cell fluorescence
DAM: Disease-associated microglia
DAMP: Damage-associated molecular pattern
EV: Extracellular vesicle
GSDMD: Gasdermin D
GSH: Glutathione
GSHee: Glutathione ethyl ester
LDH: Lactate dehydrogenase
LPS: Lipopolysaccharide
MGnD: Microglial neurodegenerative
MSU: Monosodium urate crystals
MDP: Muramyl dipeptide
NGF: Nerve growth factor
NT3/4: Neurotrophin 3/4
NLRP1/3: NLR family pyrin domain containing 1/3
PAMP: Pathogen-associated molecular pattern
