# Supplementary material for: Inflammasome activation under high cholesterol load triggers a protective microglial phenotype while promoting neuronal pyroptosis
Source: Transl Neurodegener. 2023 Mar 9;12:10. doi: 10.1186/s40035-023-00343-3 (PMC9996936; doi:10.1186/s40035-023-00343-3)
Supplement: Supplementary file 1 — Additional file 1. Fig. S1 EM of Aβ oligomers. Fig. S2 Mitochondrial GSH levels in SH-SY5Y cells. Fig. S3 Cell viability of SH-SY5Y cells after bacterial endotoxin49exposure. Fig. S4 Primary cortical neurons from SREBF2 mice show enhanced expression of inflammasome-related proteins and CASP-1 activation after Aβ exposure for 24h. Fig. S5 Uncropped scans of western blots included in Fig. 1. Fig. S6 Uncropped scans of western blots included in Fig. 4 and Fig. 5. [file 40035_2023_343_MOESM1_ESM.docx]

**INFLAMMASOME ACTIVATION UNDER HIGH CHOLESTEROL LOAD TRIGGERS A PROTECTIVE MICROGLIAL PHENOTYPE WHILE PROMOTING NEURONAL PYROPTOSIS**

Cristina de Dios^1,2,3^, Xenia Abadin^1^, Vicente Roca-Agujetas^1,2†^, Marina Jimenez-Martinez^1#^, Albert Morales^1^, Ramon Trullas^1,2^, Montserrat Mari^1^, Anna Colell^1,2*^

**Supplementary methods:**

***Electron microscopy (EM)***

The oligomer sample (initial Aβ concentration: 100 μmol/L) was diluted 1:10 in DMEM/F-12 medium and 30 μl was applied on a glow-discharged carbon-coated copper grid (400 mmesh). After 30 min of absorption excess liquid was removed using filter paper after which the samples were dried. The samples were then negatively stained with 2 % (w/v) phosphotungstic acid pH 6.8 and examined using J1010 (Jeol) microscopy coupled with a CCD Gatan Orius camera (Digital Micrograph), operating at an excitation voltage of 60 kV.

***CASP1 activity***

Cells (10^7^ cells/ml) were lysed in 20 mM HEPES, pH 7.0; 100 mM NaCl, 10 mM DTT, 1 mM EDTA, 0.1% CHAPS, and 10% sucrose, containing a protease inhibitor cocktail. The cell suspension was frozen and thawed three times and then homogenized by 10 strokes in a glass homogenizer. After clearing the extracts at 15,000*g* for 10 min, 100 μg cell lysate was added to the caspase assay buffer (100 mM HEPES, pH 7.2; 10% sucrose, 0.1% CHAPS, 1 mM Na-EDTA, 2 mM DTT) containing 0.1 mM of the CASP1 substrate Ac-WEHD-AMC. The enzymatic reaction was carried out in black 96-well plates and fluorochrome release was monitored at 37ºC for 30 min (Ex: 385 nm and Em: 460 nm).

**Supplementary figures:**


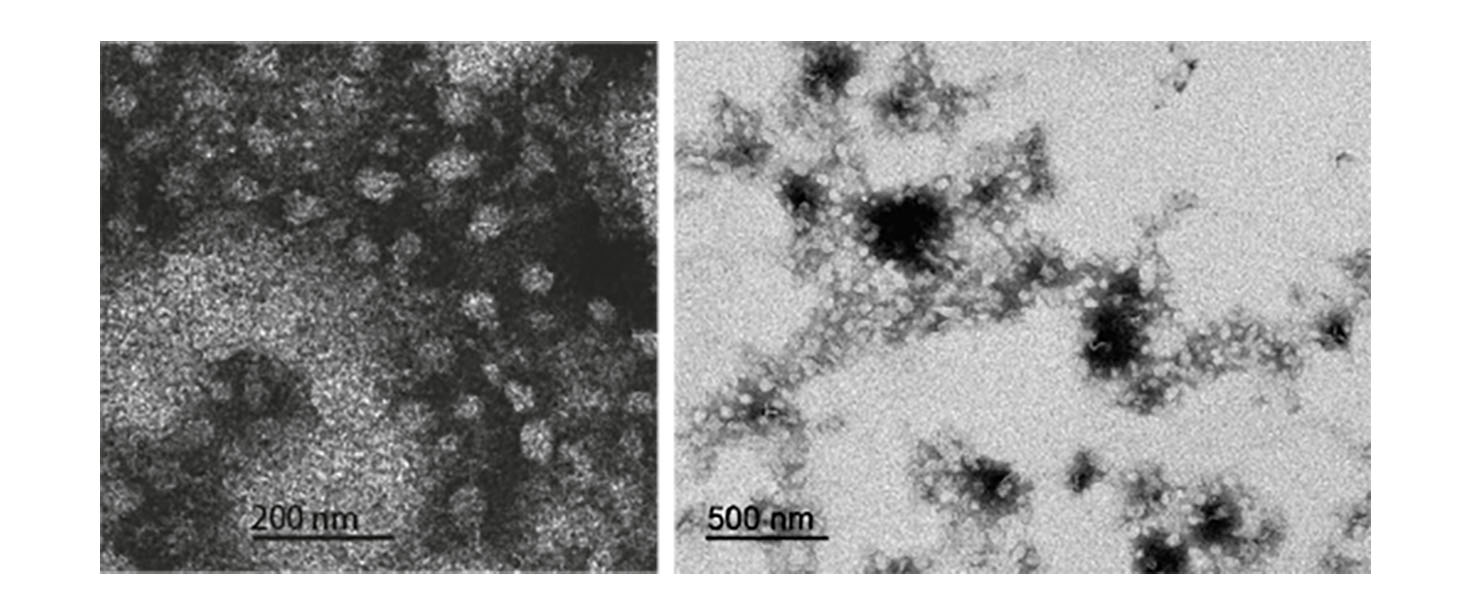


**Fig. S1** Electron microscopy (EM) of small Aβ oligomers. Aβ1-42 oligomers were produced by incubating monomers (100 μM) in DMEM medium at 4 ºC for 24 h.

**
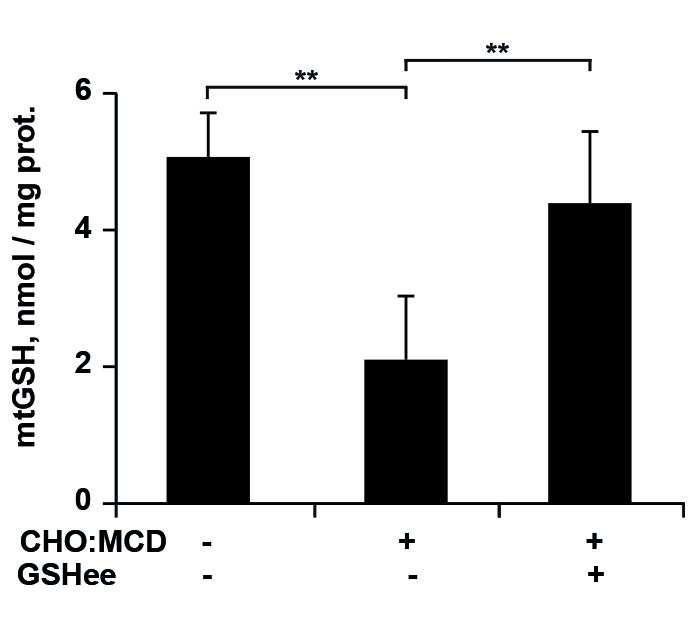
**

**Fig. S2** Mitochondrial GSH levels in SH-SY5Y cells. Cells were treated with the CHO:MCD complex for 1 h and allowed to recover for 4 h. Cells were then incubated with GSH ethyl ester (GSHee, 4 mM) for 30 min prior to cell fractionation and GSH analysis (*n* = 4-5 independent experiments). One-way ANOVA followed by the Tukey-Kramer test was applied to calculate statistical significance (** p ≤ 0.01).


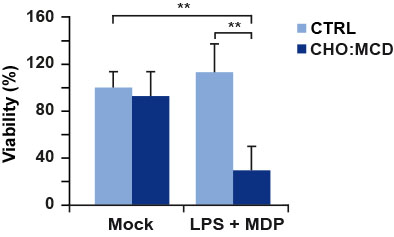


**Fig. S3** Cell viability of SH-SY5Y cells after bacterial endotoxin exposure. Cells were cholesterol-enriched by incubation with the CHO:MCD complex for 1 h and after 4 h of recovery, they were stimulated with LPS (10 μg/ml) plus MDP (10 μg/ml) for 16 h. Cell viability was assessed by trypan blue exclusion and expressed as % relative to mock cells (n = 6 independent experiments). One-way ANOVA followed by the Tukey-Kramer test was applied to calculate statistical significance (** p ≤ 0.01).


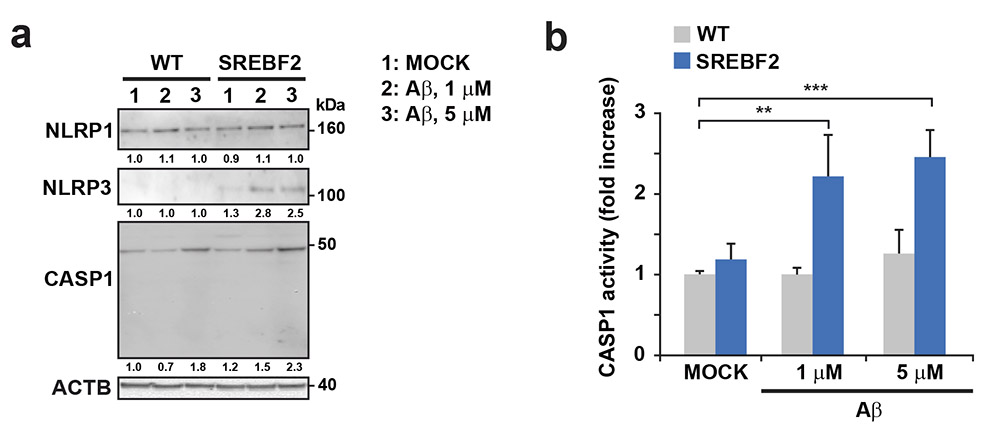


**Fig. S4** Primary cortical neurons from SREBF2 mice show enhanced expression of inflammasome-related proteins and CASP-1 activation after Aβ exposure for 24h. **(a)** Western blot analysis of NLRP1, NLRP3 and CASP1 in cellular extracts from WT and SREBF2 cells. Optical density (O.D.) values of the bands representing the specific protein immunoreactivity were normalized to ACTB/actin β staining. **(b)** CASP1 activity assessed by Ac-WEHD-AMC cleavage in untreated and Aβ-treated cell lysates. The maximal slope of arbitrary fluorescence units (AFU) versus time was calculated for each sample (*n* = 3). Results are expressed as the fold increase in activity relative to values in untreated WT cells. One-way analysis of variance followed by the Tukey-Kramer test was applied to calculate statistical significance (** p ≤ 0.01, *** p ≤ 0.001).


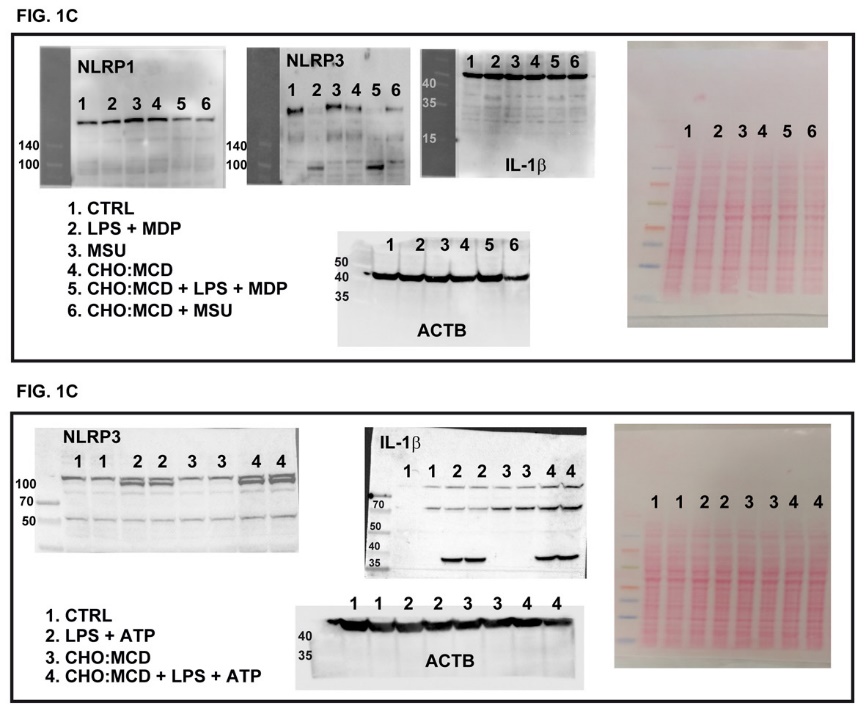


**Fig. S5** Uncropped scans of western blots included in Fig. 1


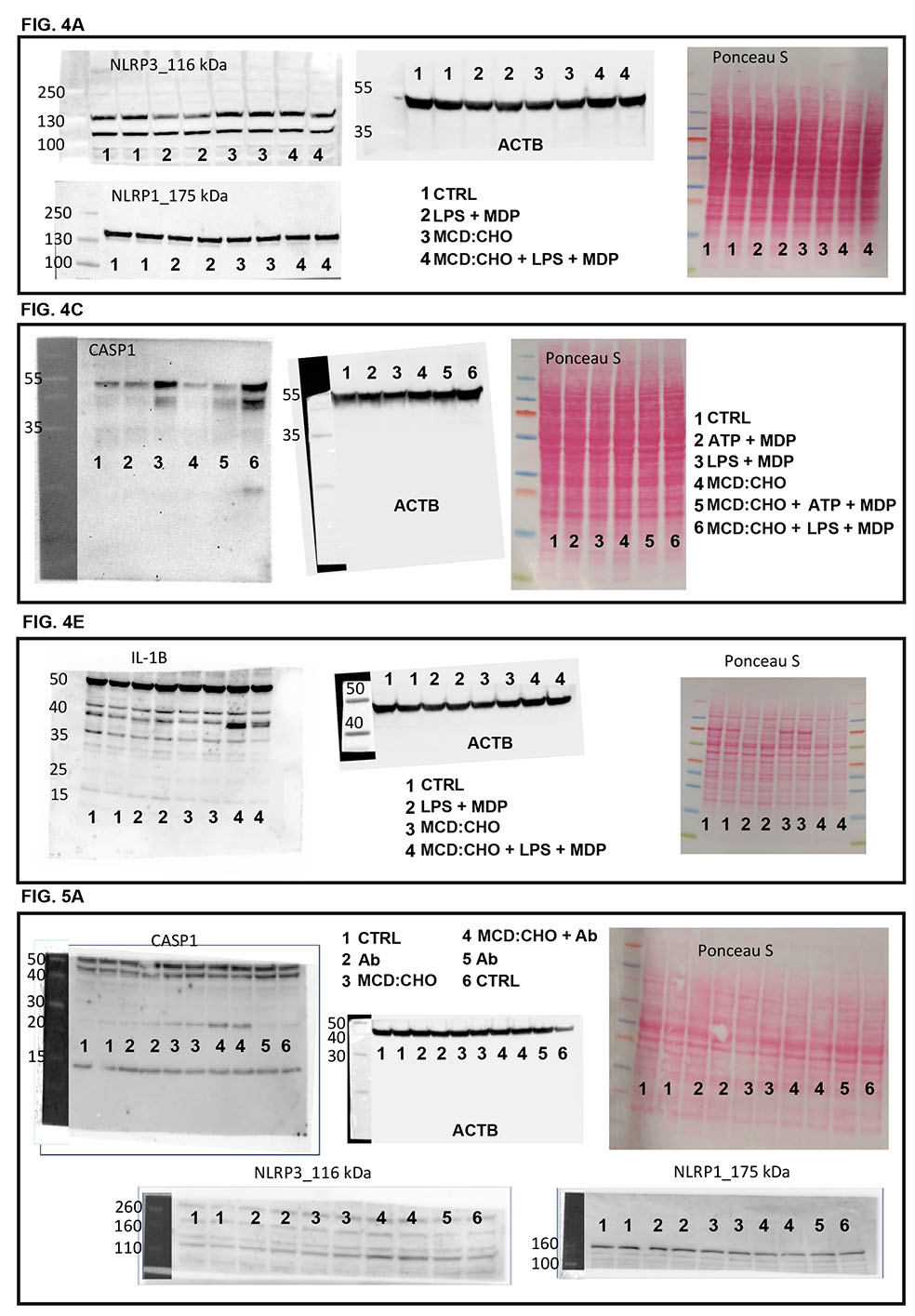


**Fig. S6** Uncropped scans of western blots included in Fig. 4 and Fig. 5
